# Thermoelectric Limitations of Graphene Nanodevices at Ultrahigh Current Densities

**DOI:** 10.1021/acsnano.3c12930

**Published:** 2024-04-19

**Authors:** Charalambos Evangeli, Jacob Swett, Jean Spiece, Edward McCann, Jasper Fried, Achim Harzheim, Andrew R. Lupini, G. Andrew D. Briggs, Pascal Gehring, Stephen Jesse, Oleg V. Kolosov, Jan A. Mol, Ondrej Dyck

**Affiliations:** † Department of Materials, University of Oxford, Oxford OX1 3PH, U.K.; ‡ Physics Department, Lancaster University, Lancaster LA1 4YW, U.K.; § IMCN/NAPS, Université Catholique de Louvain (UCLouvain), Louvain-la-Neuve 1348, Belgium; ∥ Center for Nanophase Materials Sciences, Oak Ridge National Laboratory, Oak Ridge, Tennessee 37830, United States; ⊥ School of Physics and Astronomy, Queen Mary University of London, London E1 4NS, U.K.

**Keywords:** graphene, high current density, scanning thermal microscopy, scanning transmission electron microscopy, Joule heating, Peltier effect, Seebeck coefficient

## Abstract

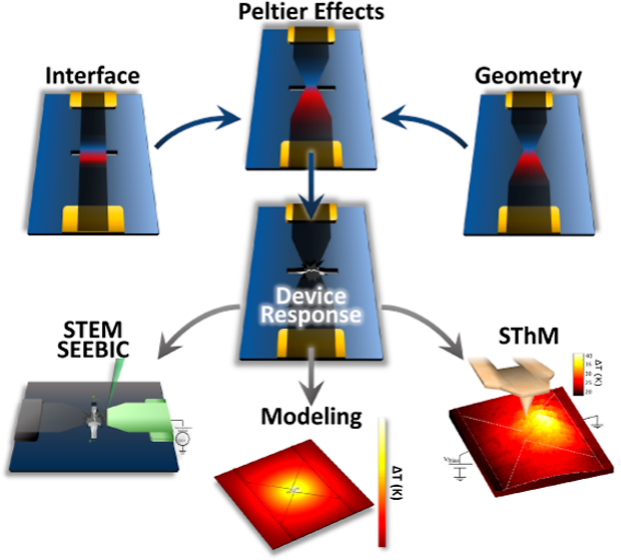

Graphene is atomically thin, possesses excellent thermal conductivity, and is able to withstand high current densities, making it attractive for many nanoscale applications such as field-effect transistors, interconnects, and thermal management layers. Enabling integration of graphene into such devices requires nanostructuring, which can have a drastic impact on the self-heating properties, in particular at high current densities. Here, we use a combination of scanning thermal microscopy, finite element thermal analysis, and *operando* scanning transmission electron microscopy techniques to observe prototype graphene devices in operation and gain a deeper understanding of the role of geometry and interfaces during high current density operation. We find that Peltier effects significantly influence the operational limit due to local electrical and thermal interfacial effects, causing asymmetric temperature distribution in the device. Thus, our results indicate that a proper understanding and design of graphene devices must include consideration of the surrounding materials, interfaces, and geometry. Leveraging these aspects provides opportunities for engineered extreme operation devices.

## Introduction

Graphene exhibits a number of physical properties that make it attractive for incorporation into modern electronic devices. Among them are high charge carrier mobility,^1,2^ the linear dispersion of Dirac fermions,^3^ high mechanical strength,^4^ and superior heat transport properties.^5^ Efforts toward integration with semiconductor fabrication have explored wafer-scale integration,^6,7^ electronic interconnects,^8−10^ photonic and optoelectronic device applications,^11^ nanopore sensing,^12,13^ and high-frequency transistors,^14,15^ among many others.

One of the most attractive properties of graphene for nanoelectronic and power electronic applications is the high current carrying capacity of this material arising from electron and phonon ballistic transport.^16−21^ Coupled with the high thermal tolerance (*i.e.*, high sublimation or melting point of 4100–4600 °C^22,23^), graphene can support a large electron current density without failure.^24,25^ However, when constructing a graphene-based device, the surrounding materials must also be taken into account. It has been noted that the substrate, for example, plays a significant role in the graphene conductivity due to the introduction of scattering centers external to the graphene itself.^26,27^ However, to date, there have been limited studies on how substrates tolerate the extreme temperature from Joule heating that can be reached when passing ultrahigh current densities through graphene. This is an important consideration since ultrahigh current densities result in significant Joule heating and thermoelectric effects^28^ that may result in damage to the substrate, or other materials in contact with the graphene,^28^ and therefore limit the practical current densities that can be obtained in these devices.

Here, we examine the effects of ultrahigh current densities in graphene nanodevices and resulting substrate damage to find the limits of extreme operation and how design considerations play a role. To do this, three device designs were chosen: (i) a suspended graphene ribbon, (ii) a graphene bowtie supported on a thin (20 nm) SiN_*x*_ substrate, and (iii) a suspended graphene bowtie. These three device designs allow for independent examination of interfacial effects, geometric effects, and the combined effect of both designs. We used scanning transmission electron microscopy (STEM) to study these devices at ultrahigh current densities, which allows for noncontact examination of device operation with high resolution. In particular, we use STEM imaging, electron energy loss spectroscopy (EELS), and secondary electron e-beam-induced current (SEEBIC) imaging^29−31^ to directly observe the breakdown modes of the three device geometries. Significant damage to the substrate was observed when operating in an ultrahigh current density regime as a result of the generated heat. We further employed scanning thermal microscopy (SThM) to explore thermal generation resulting from Joule heating, thermoelectric effects, and heat transport in graphene at low bias currents for the suspended bowtie device which combines the interface and geometrical effects of the other two designs. The results are then used to model the three designs with finite element analysis (FEA). Significant Joule heating and Peltier heating and cooling effects were observed, resulting from the graphene geometry and its interaction with surrounding interfaces. The observed damage at ultrahigh current density is a result of Joule heating that is spatially modified and enhanced by thermoelectric effects in these devices.

## Results and Discussion

**Figure 1:**
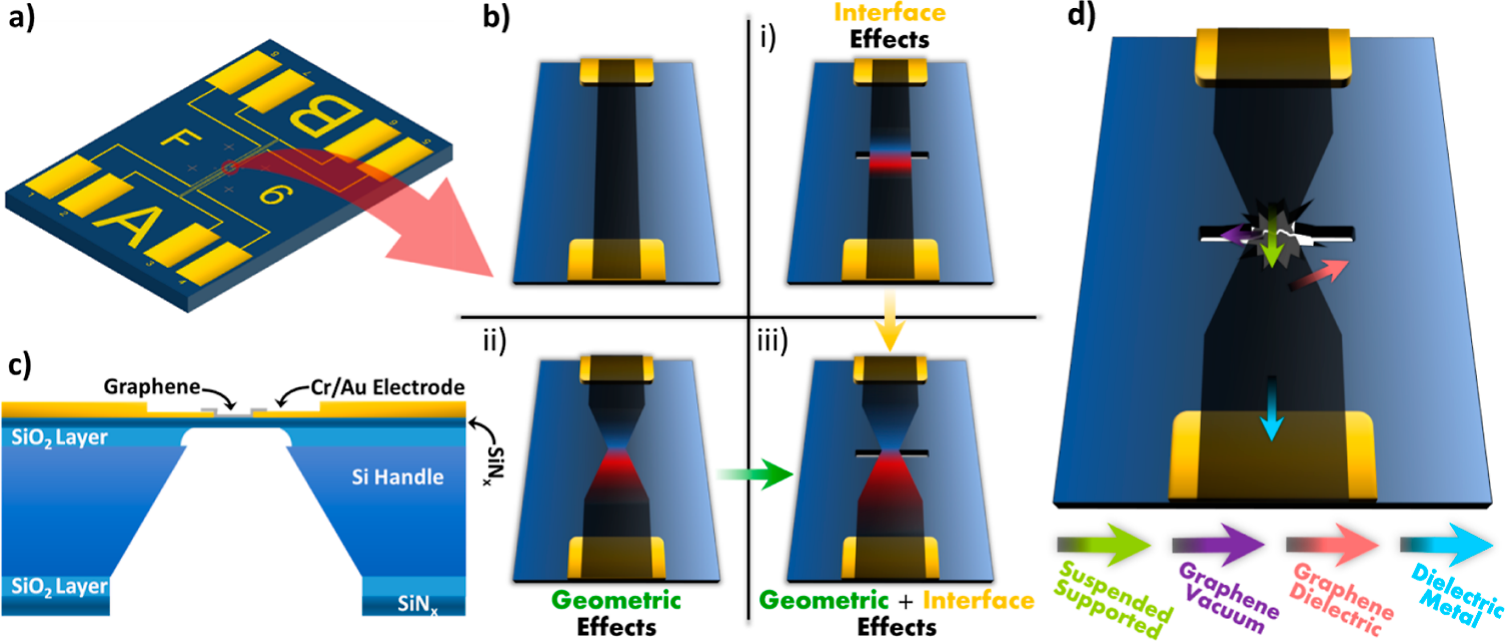
Schematic overview of investigated devices. (a) Overview of the device design. Outer and inner pairs of electrodes correspond to different (redundant) devices that can be operated on the same chip. (b) Depiction of the three device designs (i–iii) and the base graphene nanoribbon. (c) Schematic cross-section detailing the various layers of the device around the aperture. (d) Schematic diagram illustrating the different types of interfaces.

Schematics of the investigated device geometries are shown in Figure 1a–c. The basic device geometry consists of a 20 nm thick Si-rich SiN_*x*_ membrane suspended above a 1000 nm thick SiO_2_ layer on a Si substrate (Figure 1c). Metal electrodes (Cr/Au) were patterned on the surface of the SiN_*x*_ membrane *via* photolithography and electron beam lithography (EBL) (Figure 1a), which act as source–drain electrodes for the graphene nanodevices. We examined three device designs to understand the role of graphene geometry and interfacial interactions on the thermoelectric effects of the devices: (i) a straight graphene ribbon on a SiN_*x*_ membrane with an aperture creating a suspended region [Figure 1b(i)], (ii) a graphene bowtie supported by the SiN_*x*_ membrane [Figure 1b(ii)], and (iii) a graphene bowtie on a SiN_*x*_ membrane with an aperture creating a suspended region [Figure 1b(iii)]. The aperture dimensions, along and perpendicular to the device direction, were chosen depending on the requirements of each experimental technique. For the STEM imaging, a wider electron transparent window with a completely suspended central region is necessary to facilitate imaging of graphene. In contrast, for SThM, a design with a narrower window was used to support graphene from all four sides and avoid any damage of the graphene by the SThM tip. As shown in the Supporting Information, the width does not affect the thermal phenomena occurring in the device. For the STEM experiments, the dimensions were 700 × 300 nm and 6000 × 200 nm for designs (i) and (iii), respectively, and for the SThM experiments, the dimensions were 100 × 600 nm for design (iii).

These designs represent alterations to a plain supported nanoribbon, which captures the effects of the geometry and interfaces. The plain nanoribbon can support increasing current density until the breakdown of graphene^24,32^ or the electrodes reach their melting temperature, mainly the drain, as suggested by our FEA calculations (see Supporting Information). Geometric and interfacial thermoelectric effects move the region of extreme temperature away from the contacts. A detailed description of the device fabrication procedure is given in the Experimental Section.

### *In Situ* STEM Measurements

We began by using *operando* STEM to allow noncontact, fast imaging observation of the devices during operation in the extreme current regimes. High-temperature damage of graphene and the substrate was monitored through real-time STEM imaging while operating the device at high current densities. The spatial correlation and degree of substrate damage provide indirect information on the temperature distribution of the device. Since we operate in ultrahigh vacuum at constant pressure, the origin of the damage is sublimation driven by temperature. We locate and visualize the graphene devices using high-angle annular dark field (HAADF) and SEEBIC imaging and then Joule heating the devices while continuously recording HAADF images. We ramp up the voltage at a rate of 7.5 V s^–1^ to a predetermined threshold voltage and then quickly ramp back to 0 V (2000 V s^–1^). The threshold voltage was typically 0.4 V for the first cycle and increased by 0.1 V for each cycle until the breakdown of the graphene. After each cycle, we measured the device electrical resistance. Alternatively, in some cases, we applied a constant high voltage for a specified amount of time, which does not necessarily cause graphene breakdown.

**Figure 2:**
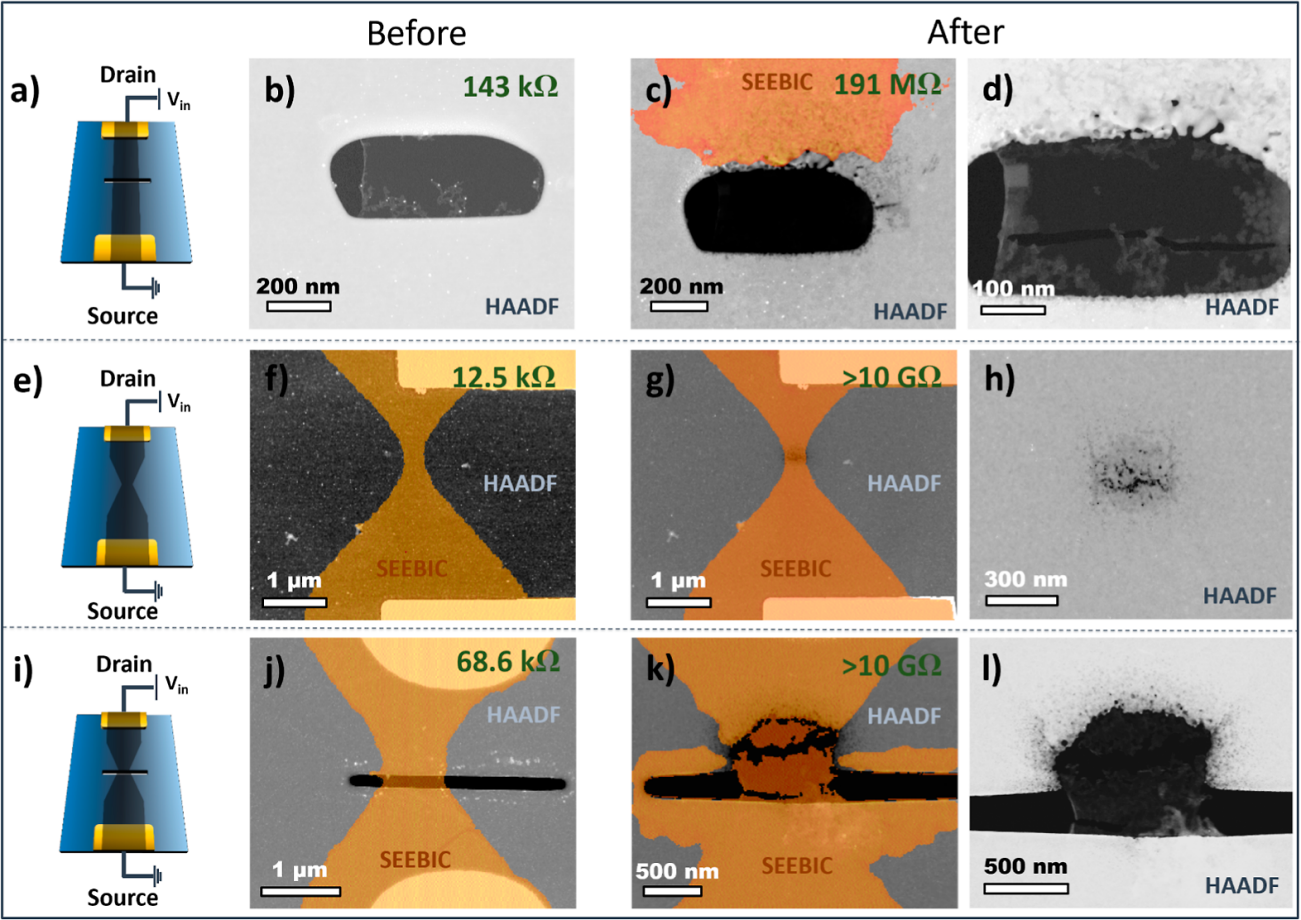
Examples of various graphene device configurations before and after operation in high current density conditions. (a,e,i) show a schematic representation of each device configuration. (b,f,j) show HAADF images of each configuration prior to operation. The orange overlays show SEEBIC data which was simultaneously acquired with the HAADF images highlighting the conductive regions of the sample. (c,g,k) show HAADF/SEEBIC images of the devices after operation to failure. (d,h,l) show magnified HAADF images of the burned areas. Note that for device shown in (a,e), voltage ramps of increasing voltage up to breakdown were applied, with a maximum value being 4.8 and 4.9 V, respectively. For device (i), a constant voltage was applied up to failure. (f–h) Adapted by permission from John Wiley and Sons: Small Methods (ref (31)), copyright 2022. (j–l) Adapted from Oxford University Press: Microscopy and Microanalysis ref (34), copyright 2022.

The three device configurations were examined using STEM, and they are summarized in Figure 2. Figure 2a,e,i shows a schematic of the designs with the location of the source and drain used during device operation. Figure 2b,c,f,g,j,k shows the composite HAADF/SEEBIC images as well as the resistance of the device before and after breakdown, respectively. Figure 2d,h,l shows a magnified HAADF view of the breakdown site of the device. The creation of the composite HAADF/SEEBIC images is described in full in the Supporting Information. Briefly, HAADF and SEEBIC channels were recorded simultaneously, and the nonconductive regions of the SEEBIC channel (*i.e.*, the darker areas) were removed from the image using the trainable Weka segmentation plugin for ImageJ to discriminate between regions.^33^ The conductive regions in the SEEBIC image were then tinted orange and overlaid on the HAADF image to highlight the location of the graphene.

The damaged location from the suspended ribbon was imaged after graphene breakdown and is shifted toward the drain side of the device (see Figure 2i–l). The graphene on the source side became electrically disconnected from the contacts and thus did not appear in the SEEBIC image. Interestingly, some parts of graphene at the edges of SiN_*x*_ sublimated. For the supported bowtie device, Figure 2i–l, again we observe the mass loss from SiN_*x*_ to be shifted from the narrowest graphene region toward the drain (see Figure 2d). The asymmetry is more evident for the suspended bowtie geometry when driving the device to failure. The drain side of the device again exhibits the majority of the sublimation-driven mass loss from the SiN_*x*_ substrate. The graphene interface with the substrate on the source side shows no mass loss, suggesting a substantial temperature difference between the two sides during the breakdown process. It should be emphasized that these two sides are only 250 nm apart and are thermally connected by the graphene. Additional examples showing asymmetric breakdown can be found in the Supporting Information. Examining the SEEBIC overlay, we observe a high conductivity region surrounding the entire aperture after the device is driven to failure. This rather unexpected observation is possibly the result of the thermally graphitized surface hydrocarbons. EELS spectra of this location can be found in the Supporting Information. Detailed investigation of this phenomenon is outside the scope of this article.

### SThM Measurements and FEA Analysis

**Figure 3:**
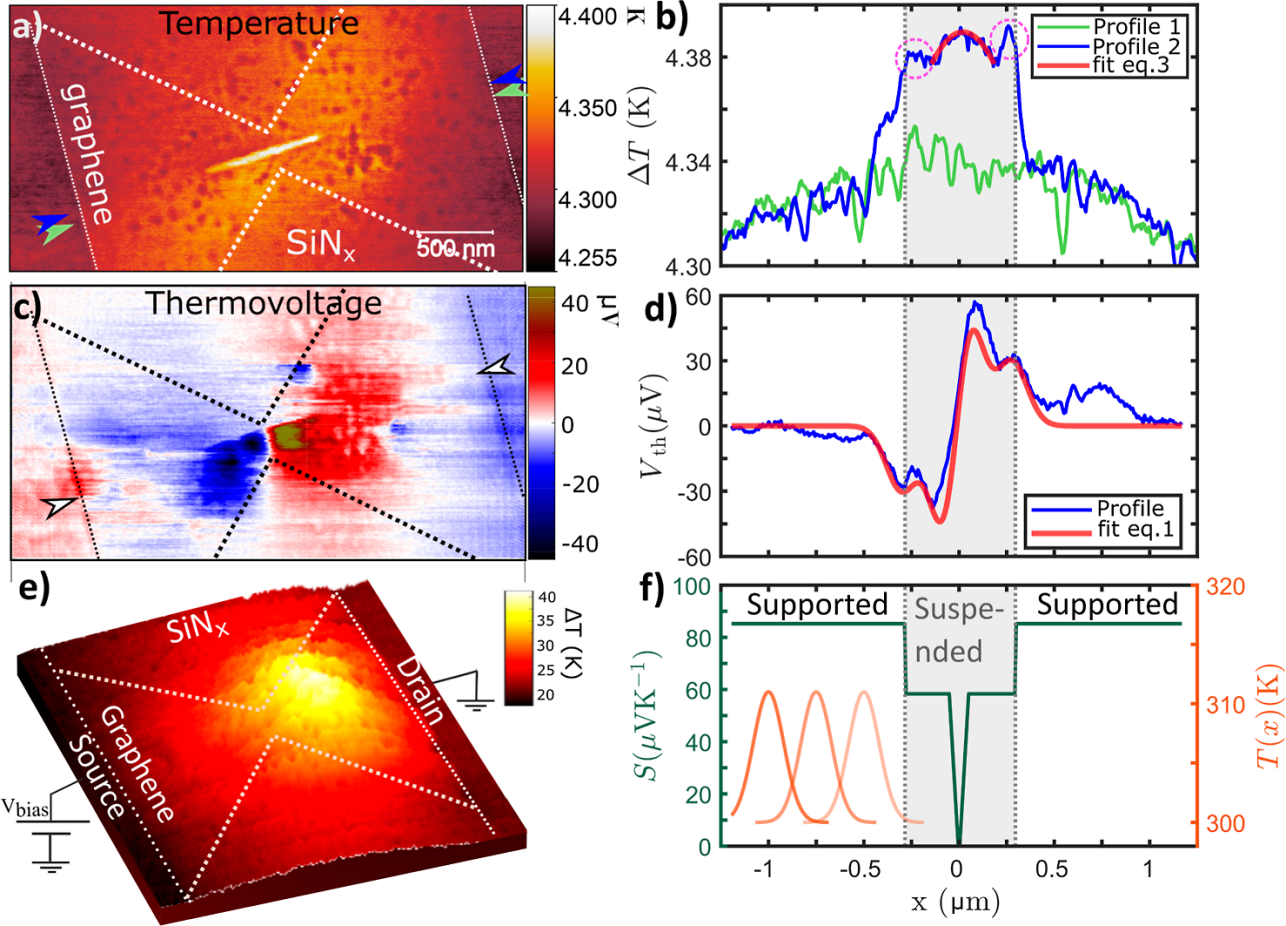
Temperature (a) and thermovoltage (c) maps of a bowtie graphene device on the SiN_*x*_ membrane with a longitudinal slit of dimensions 100 × 600 nm (device 1). Note that for (a), the applied power on the device was relatively low at *P*_dev_ = 19 μW (*V*_bias_ = 2 V), and for (b), the excess heat temperature of the tip was Δ*T*_tip_ = 11 K. Profiles of temperature (b) and thermovoltage (d), as extracted from images (a,c), respectively, along and next to the slit, as denoted by the arrows. The red curve labeled “fit” in (b) is the fitting of eq 4 and in (d) is the fitting of eq 1. The mini-peaks of the temperature profile in the suspended region are encircled by magenta dotted line regions. (e) 2D-temperature map of a similar device (device 2) at a higher power of *P*_dev_ = 50 μW (*V*_bias_ = 4 V). (f) Seebeck coefficient profile obtained by fitting [red line in (d)] the thermovoltage profile with the developed model (left *Y*-axis). Gaussian temperature spot created by the hot tip while moving along the device (right *Y*-axis) for 3 different tip positions. The shaded portion of (d,e,f) corresponds to the suspended region over the longitudinal slit.

To understand the origin of the high current density breakdown process, we employ SThM, at room temperature in high vacuum (10^–6^ Torr), to measure the thermoelectric and Joule heating effects. We focus on the suspended bowtie device because it combines both geometry and interface-related thermoelectric effects. Figure 3a,e shows temperature change maps for low-biased devices 1 and 2, respectively (see Supporting Information for details on how the maps were obtained). The temperature distribution along the device is asymmetric with the higher absolute temperature shifted toward the device’s drain. This is in agreement with the previously described observed damage with STEM. In contrast to our previously investigated bowtie-shaped device on SiO_2_,^28,35^ the temperature in the supported regions increases not only at the narrowest region of the graphene but in the surrounding regions as well. This is due to the lower heat dissipation to the thin SiN_*x*_ substrate compared to that of thicker substrates. Furthermore, the temperature rise in the suspended region is greater than in the supported region, despite its lower thermal resistance (see Supporting Information for the thermal resistance map). Note that some local features (spots with lower temperature) appearing in the temperature map of device 1 are due to convolution with the SiN_*x*_ rough topography (see Supporting Information for topography maps). Furthermore, the temperature increase in the suspended region of device 2 is not visible in the temperature map due to the larger (comparable to the slit width) contact radius of the tip used for this device (see Supporting Information for a discussion on the tip–sample contact area effect on SThM imaging). Note that the absolute temperatures are quite low due to the small bias voltages applied, to avoid breakdown or alteration of the device, while recording temperature maps.

To better understand the effect of suspending the graphene on the resulting temperature, we plot the temperature profiles along the aperture from the source to the drain of the device and near the aperture for comparison (Figure 3b blue and green lines, respectively). The profile for the completely suspended part has a parabolic shape with an asymmetry toward the drain. The supported part of the profile along the aperture follows the same trend. At the boundaries of the supported and suspended regions, a jump in temperature is observed, accompanied by mini-peaks just inside the suspended region (see encircled by magenta dotted line regions). Away from these mini-peaks, in the suspended graphene, the temperature exhibits a parabolic profile, as shown in the fit in Figure 3b.

The features of the temperature maps discussed above have their origins in different phenomena, including Peltier heating/cooling, Joule heating, and heat transport mechanisms.^36^ To distinguish between thermoelectric effects from Joule heating and thermal transport-related effects, we mapped the thermovoltage (*V*_th_) generated in the device in contact with the heated SThM tip using the scanning thermal gate microscopy (STGM) approach^37^ and extracted the Seebeck coefficient (*S*) of the device. The Seebeck coefficient can then be used as an input for FEA calculations of the Peltier and Joule heating contribution to the temperature. *V*_th_ is defined as the voltage built at the source and drain of the device for a given *S* due to a temperature increase caused by the SThM tip. Therefore, by raster scanning the device with the hot tip and recording the voltage drop at the contacts, we map the *V*_th_ caused by the local changes of the Seebeck coefficient (see Figure 3c). We observe a decrease/increase at the left/right side of the graphene constriction, respectively, for both suspended and supported regions, caused by a change in *S* due to the decreased width of graphene.^28^ At the gold contact borders, an increase/decrease in the thermovoltage is also observed, albeit with lower intensity, as a result of different *S* of gold and graphene. Additional small variations in the thermovoltage map are related to the local variation of the Seebeck coefficient of graphene^37^ or with the substrate roughness which results in some parts of the graphene being poorly attached to the substrate.

To get the absolute *S*, we plot the profile *V*_th_ along the aperture on SiN_*x*_ and follow the same procedure as ref (28) by fitting it with a position-dependent Seebeck coefficient model. The *V*_th_ profile along the device shows two minima in the left-hand side of the constriction and two maxima on the right-hand side (Figure 3d). The absolute minimum and maximum values are related to changes in the Seebeck coefficient due to the graphene geometry. The decrease in graphene width reduces the electron mean free path (EMFP), which in turns reduces the local Seebeck coefficient by several orders of magnitude.^28^ More specifically, the EMFP of graphene at room temperature is typically on the order of hundreds of nanometers and significantly reduces by scattering at defects such as the rough edges of graphene.^38^ This scattering effect dominates as the graphene width decreases (in the middle of the bowtie device), creating a position-dependent EMFP and Seebeck coefficient. Similar effects have been observed in metallic thin-film stripes and Au nanowires.^39,40^ We speculate that the additional minima/maxima are related to different Seebeck coefficients of the suspended and supported graphene regions. The thermovoltage is expressed as a function of the position-dependent Seebeck coefficient
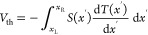
1where *S*(*x*′) is the spatially dependent Seebeck coefficient, d*T*(*x*′)/d*x*′ is the position-dependent gradient of the temperature profile due to the hot tip, and *x*_L_ and *x*_R_ are the positions of the left and right electrodes of the device. We assume temperature gradient of a symmetric Gaussian temperature hot spot caused by the hot tip while moving over the device (see Figure 3f)  with Δ*T*_tip_ being the excess temperature of the tip, which is known through the probe temperature calibration (see Supporting Information), *T*_0_ the room temperature, *x*_tip_ the position of the tip, and σ the standard deviation of the distribution. Then we use a simple phenomenological model for *S*(*x*) which accounts for the main qualitative features of Figure 3d: it decreases to zero at the narrowest part of the constriction and consists of different values *S*_0_ and *S*_1_ for the supported and suspended regions, respectively (Figure 3f)
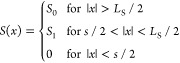
2where *L*_S_ is the width of the graphene constriction and *s* is the length of the aperture on the SiN_*x*_ substrate (for more details on the model, see Supporting Information). We fit eq 1 to the experimental thermovoltage profile (see the red line in Figure 3d) with *S*_0_, *S*_1_, and σ being the fitting parameters. The resulting values *S*_0_ = 85 μV K^–1^ and *S*_1_ = 58 μV K^–1^ are in good agreement with reported values for supported^41−43^ and suspended graphene,^44^ respectively. We attribute the higher *S* of supported graphene to doping induced by the SiN_*x*_ substrate.^45,46^ Finally, we find σ = 85 nm for the Gaussian hot spot which gives a width of Gaussian distribution of 750 nm, which explains the nonzero thermovoltage on SiN_*x*_.

When a voltage is applied to the device, such changes in the Seebeck coefficient induce Peltier heating/cooling effects. The Peltier effect is the Onsager reciprocal to the Seebeck thermoelectric effect and is the heat flow induced by the electrical current in a material *Q̇* = Π*I* with Π = *TS* being the Peltier coefficient. Therefore, we use the obtained *S*(*x*) as an input to FEA calculations of Peltier and Joule heating temperature maps of the devices for small bias currents (see Supporting Information for details on the model). In addition, to separate thermal and thermoelectric effects related with the graphene geometry and graphene interface to the surrounding materials, we model the two other device geometries imaged with STEM during ultrahigh current operation by using a spatially dependent *S*(*x*) estimated from the *S*_0_ and *S*_1_ values obtained (see Supporting Information for *S*(*x*) used for modeling each device).

**Figure 4:**
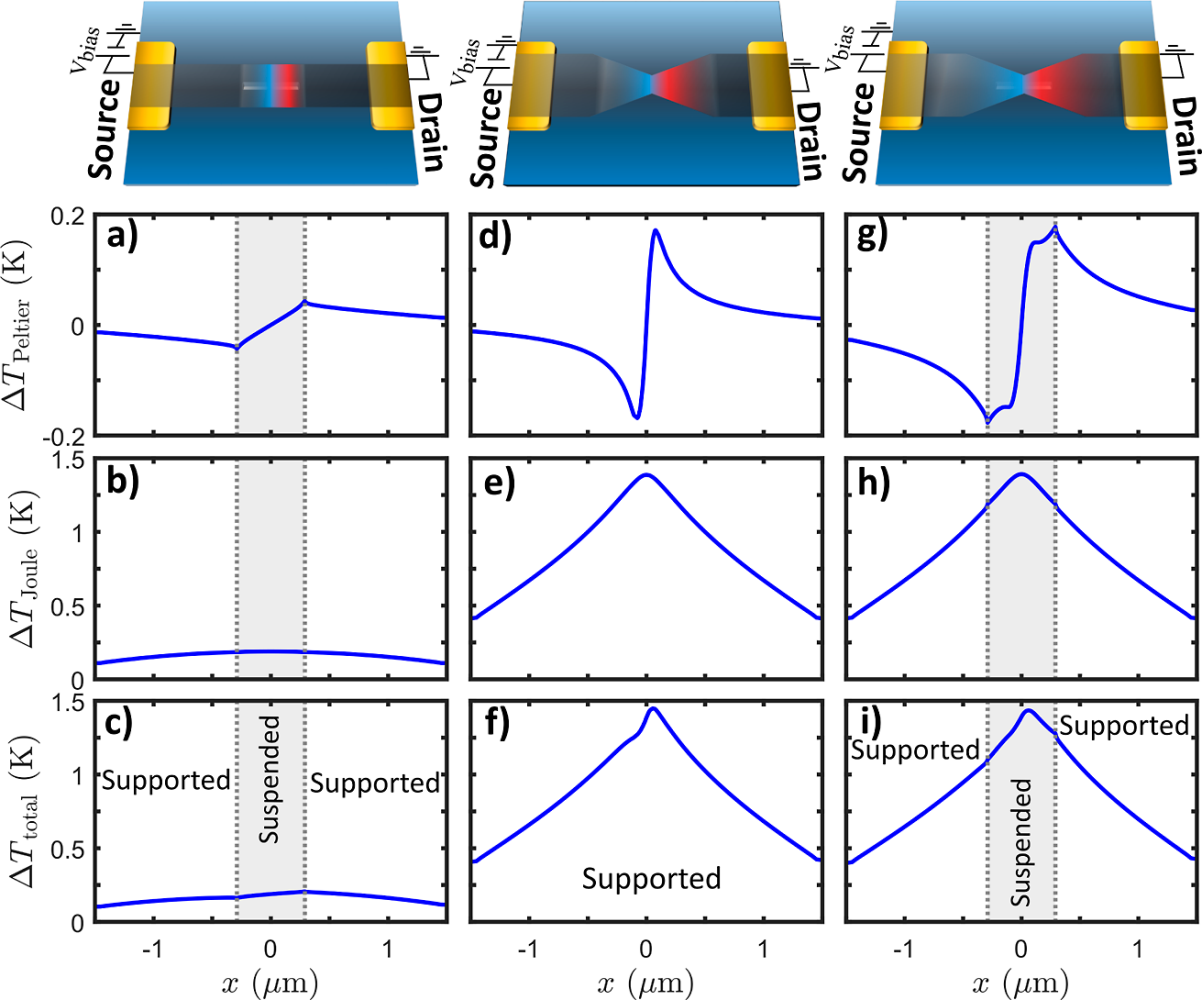
FEA results: Peltier heating/cooling, Joule heating temperature, and total temperature profiles: straight ribbon on SiN_*x*_ with an aperture (a–c), bowtie graphene (d–f) on SiN_*x*_, and bowtie graphene on SiN_*x*_ with an aperture (g–i). Note that the borders of the suspended graphene are shown with dotted gray lines.

Starting from the suspended ribbon, the interfaces introduced by the aperture cause a cooling/heating effect, with the minimum/maximum values being at the aperture edges (Figure 4a). The Joule heating is very small, as the cross-section of the device is larger, resulting in a reduced current density (Figure 4b). The asymmetric effect due to Peltier heating and cooling in the total temperature profile is notable (Figure 4c). For the supported bowtie device, the Peltier temperature shows geometrically induced cooling/heating at the source/drain side of the constriction in line with the Seebeck profile (see Figure 4d). The Joule heating on the other hand has a normal symmetric distribution with a maximum value at the center of the device (see Figure 4e); however, the absolute values are much higher for this device than for the suspended ribbon. The Peltier heating/cooling causes a small asymmetry to the total temperature profile; however, this effect is relatively small compared to the Joule heating (Figure 4f). The suspended bowtie device combines both geometric and interfacial effects. For the Peltier temperature map, symmetrical cooling/heating at the source/drain side is observed with the difference that two minima in the cooling and two maxima in the heating appear (Figure 4g). These minima/maxima are a result of geometric and interfacial effects related to the features observed in Figure 3d. Joule heating is similar in the supported bowtie device with the difference that the rate of increase is higher in the suspended region than in the supported region (Figure 4h). The total temperature, as in all of the devices, is asymmetric (see Figure 4i). The highest temperature value at the middle of the suspended bowtie is similar to that of the supported bowtie. However, comparison of absolute temperature at a point which is supported in the first device and suspended in the other is misleading due to different boundary conditions of FEA calculations of the two devices. In addition, ballistic effects are not taken into account, and the temperature values are strongly dependent on the relative values between the supported and suspended thermal conductivities chosen as input to the FEA (see Supporting Information for details).

In the Peltier temperature profiles, the higher temperatures are always toward the drain of the device. In Supporting Information, we confirmed experimentally and with FEA calculations that by swapping the drain and source, the warmer values also changed side. This is due to the dependence of the Peltier heating/cooling rate, *Q̇*, on the current and the Seebeck coefficient of the device. Specifically, the spatial temperature difference is given by^47^
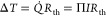
3with *R*_th_ being the graphene thermal resistance and Π = *TS* the Peltier coefficient. Thus, the Peltier temperature in the direction of the device will depend on the Seebeck profile and current direction. Therefore, by applying a positive voltage at the source and for the spatial Seebeck coefficient shown in Figure 3f, the temperature will be asymmetric with higher values toward the drain side.

Comparing the calculated temperature profile to the experimental temperature profile for the suspended bowtie device, we see that it captures the asymmetric heating and the increased temperature in the suspended region. However, the FEA results do not show any temperature jumps at the edges of the SiN_*x*_ aperture, as observed experimentally. Such temperature jumps in nanostructures are indicative of ballistic or semiballistic heat transport.^48^ The temperature at the contacts has been theoretically predicted for nanostructures regardless of the heat generation mechanism (internal or at the contacts) and are attributed to a mixing of ballistic phonons of the heat source (*i.e.*, Joule heating in the device) and the heat sink (*i.e.*, contacts) with different temperatures, which creates an internal boundary resistance. The jumps do not appear because of phonon scattering at the edges as one would assume.^44,48−50^ The phonons from the suspended region propagate ballistically with a high mean free path toward the edges, which reduces when reaching the supported region. This transition is signaled at the temperature profile of Figure 3d by the temperature mini-peaks just inside the suspended region and after the jumps at the suspended–supported boundary. In addition, the different thermal transport mechanisms in the suspended and supported graphene result in a lower measured thermal resistance in the suspended region (see Supporting Information for the relevant thermal resistance maps). One might expect the opposite since the supported region has an extra heat dissipation channel; however, the reduction of the lateral heat transport due to the thin substrate is the dominant effect.^51^ Further discussion and evaluation of the ballistic heat transport contribution can be found in the Supporting Information.

The parabolic shape of the temperature in the suspended part away from the mini-peaks is in agreement with the calculated ones. For such small bias currents, this temperature increase is mostly due to Joule heating and much less due to the Peltier effect. The temperature profile due to Joule heating is given by^49,52^
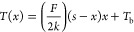
4where *T*_b_ is the temperature at the boundaries just after the jumps, *s* is the length of the device, *k* is the thermal conductivity of graphene, and *F* = *PA*^–1^ is the Joule heating generation term. Since this equation is extracted classically from Fourier’s law and is valid in the diffusive thermal transport regime, we choose to fit only the parabolic part of the temperature profile of the suspended regime (see the curve in the gray shaded part of Figure 3b) excluding the mini-peaks. This fitting gives *k* = 795 Wm^–1^ K^–1^ for the suspended graphene.

While these temperature maps are for low bias currents, for applications where high currents are desirable, it is important to know the Peltier and Joule temperatures at higher current densities. The classical electrical and thermal equations predict that the Peltier heating/cooling temperature increases linearly with the current, while Joule heating increases with the square of current. FEA calculations confirm these trends for increasing bias current (see Supporting Information for more details); however, for extremely high currents, the temperature of the whole device increases due to the boundary conditions set in the model, making it difficult to draw any conclusions. Considering these classical trends, one would expect a decreased Peltier heating/cooling relative contribution to the total temperature at higher currents. At the same time, it is to be noted that for larger total temperature elevations *T*_total_ > *T*_ambient_, while Joule retain the same square dependence on the drive current, increasing the role of the Peltier effect in heat generation (Δ*T*_Peltier_ ∼ *T*_total_ × *P*_Peltier_ ∼ *I*^3^). For graphene, the measured Peltier temperature rise at high currents has been found to have a cubic dependence with current, making its heating contribution important in this regime. This additional contribution to the Peltier effect heating can be attributed to an “electron wind” resulting from the high drift velocity of charge carriers approaching the Fermi velocity in the constriction of the device^28^ as well as to the temperature rise comparable with ∼300 K initial temperature values. Furthermore, to evaluate the contribution of the Thomson effect to the asymmetric heating, we have mapped the temperature rise for variable current through the device, with power ranging from 0.9 to 19 μW, which allows us to distinguish between the heat generation effects with different power dependences on the current. At moderate local temperature rises compared with the ambient (*T*_ambient_ ∼ 300 K) and assuming a temperature-independent Seebeck coefficient, the local temperature increase related to Peltier effect (Δ*T*_Peltier_) is linear with the current (Δ*T*_Peltier_ ∼ *P*_Peltier_ ∼ *I*) while the Joule heating is proportional to the square of current (Δ*T*_Joule_ ∼ *P*_Joule_ ∼ *I*^2^). On the other hand, for the Thomson effect is linear with the cube of current (Δ*T*_Thomson_ ∼ *P*_Thomson_ ∼ *I*^3^) as the Thomson effect is proportional to the temperature gradient and hence to the local temperature rise driven by Joule heating. By comparing the temperature rise at low heating with power on the device raising from 0.9 to 19 μW (Supporting Information, Figure S4f), it is clear that the asymmetry of the heating is at least the same if not higher at the lower power, suggesting the negligible role of the Thomson effect and, hence, low dependence of the Seebeck coefficient on the temperature.

**Figure 5:**
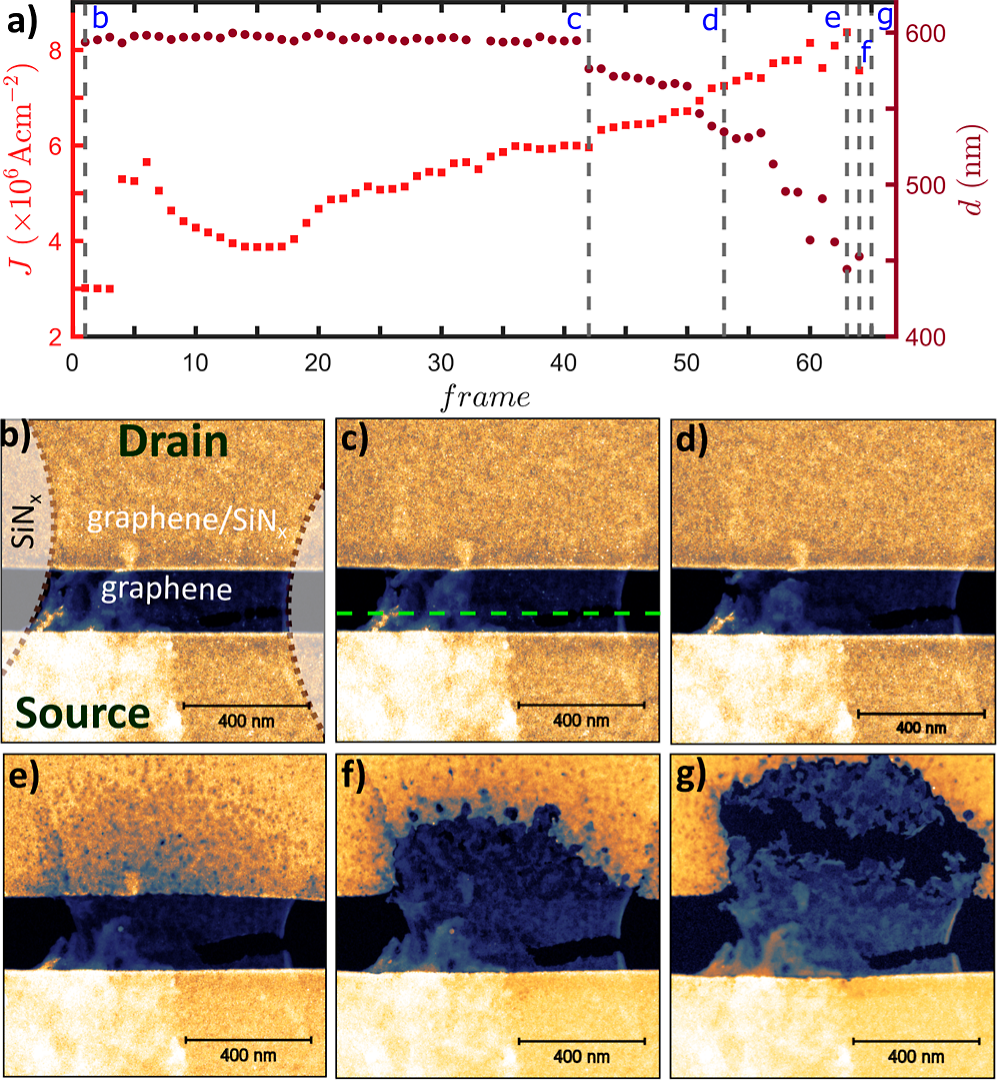
(a) Low bias current density and cross-section width evolution of the graphene device with HAADF frames up to graphene breakdown (resistance evolution can be found in Supporting Information). The total time of the process is 19.6 s. Dotted lines indicate the location of the frames presented in (b–g). Note that at frame (g), the device broke and current was no longer observed. The graphene borders are shown in image (b) with dotted lines as well as the source–drain direction. The cross-section width was measured along the green dashed line shown in image (c). Voltage applied to the device before acquiring image (c,d,e,f,g) was 3.3, 4, 4.6, 4.8, and 4.9 V, respectively. (g) Adapted from Oxford University Press: Microscopy and Microanalysis ref (34), copyright 2022.

To better understand the high current density breakdown process, we examine in greater detail the suspended bowtie device, presented in Figure 2e–h with *operando* STEM, during the breakdown process. Figure 5b–f shows images acquired during the voltage ramp process for this device, while Figure 5a shows the current density of a graphene cross-section at a low bias of width *d* (as drawn with a green dashed line in Figure 5c) with respect to the HAADF frame number. The current density increases during the first cycles and then decreases up to a minimum value; however, no visible changes in the imaged region of the devices or the cross-section width are observed in this region. The minima in current density are followed by an increase, and some cracks in the suspended graphene are created (see Figure 5d) and the corresponding *d* starts to decrease. We believe that the initial variations of current density are related to competitive effects, such as sublimation of polymer residues left from the lithography process either on the graphene or at the graphene–gold contact interface and an increase in graphene’s disorder due to the relatively high applied voltages.^35^ In the final cycles before complete breakdown, the current density increases. The existing cracks propagate and the substrate starts degrading at the drain side of the device (see Figure 5e). Specifically, the edge of the SiN_*x*_ aperture on the drain side is damaged first followed by the neighboring areas, and finally, the whole area below the graphene sublimates (see Figure 5f). At the same time, the SiN_*x*_ on the source side of the device is only minimally damaged. The asymmetric damage of the substrate toward the drain is in line with the asymmetric heating of the device observed with the SThM and FEA calculations. Degradation of SiN_*x*_ at the window edges is in good agreement with the SThM temperature map and the increased temperature at the edges. Finally, the graphene breaks, and a gap of a few tens of nanometers is created (see Figure 5g). The asymmetric damage of SiN_*x*_ observed in STEM experiments is a result of sublimation due to Joule heating and Peltier heating/cooling, causing the asymmetry. We exclude the possibility that this asymmetry arises from the thermal expansion of SiN_*x*_ since the main component of temperature increase at high current densities is the Joule heating, which is symmetric in the plane of the device. The imbalance of the temperature distribution created by the Peltier heating/cooling is unlikely to cause the asymmetric breakdown. On the other hand, the different expansion coefficients (positive for SiN_*x*_ and negative for graphene) accelerate the breakdown of graphene itself and the propagation of the cracks as can be seen in the video (as formed by joining all the frames) recorded with STEM during breakdown (see Supporting Information).

**Figure 6:**
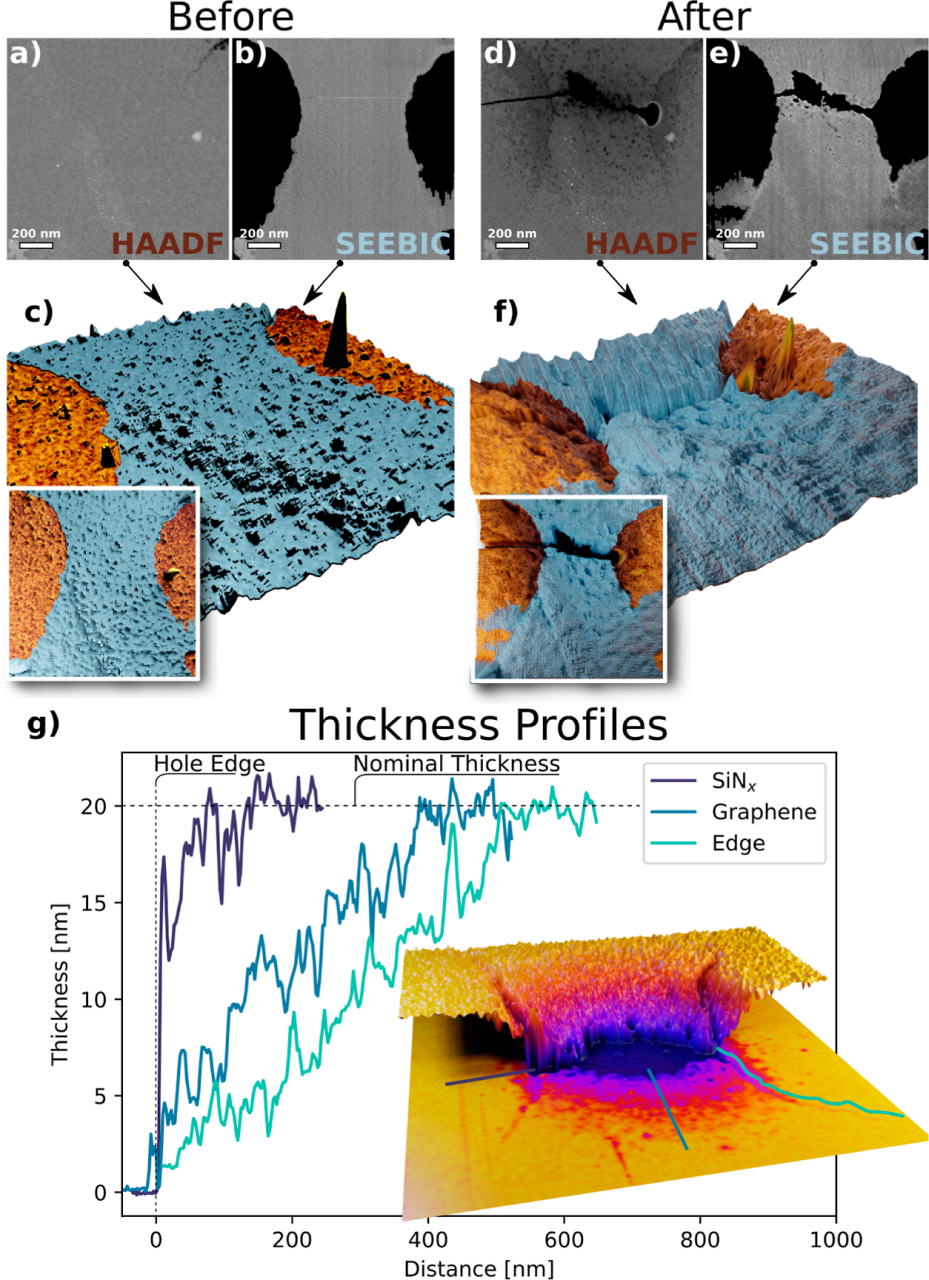
Visualization of the mass loss in the burned area. HAADF intensity before (a) and after operation (d) can be used as a proxy for sample thickness. A simultaneously acquired SEEBIC signal can be used to reveal the location of the graphene through conductivity (b,e). Combined signals in top-down and angled views of the sample using a color-coded 3D-surface plot (c,f). A second example is shown in (g). The HAADF image, inset, was converted to artificially colored thickness and displacement maps. Intensity profiles were taken from the regions indicated by the color-coded lines. The profiles were adjusted to align at the hole edge. We see extended mass loss at the edges of the graphene nanoribbon. (d,e) Adapted from Oxford University Press: Microscopy and Microanalysis ref (34), copyright 2022.

In Figure 6, we examine the SiN_*x*_ mass loss surrounding the heated region of supported bowtie graphene more closely. The intensity of the HAADF image scales approximately linearly with thickness in thin amorphous materials and can thus be interpreted as an approximate thickness map. Our SiN_*x*_ window is 20 nm thick, and using the vacuum intensity as a zero reference, we can scale between these two references to visualize the mass loss more intuitively. Figure 6a,b,d,e shows pairs of simultaneously acquired HAADF and SEEBIC images (a,b) before operation and (d,e) after device failure. The SEEBIC images have been filtered for noise and the nonconductive regions set to black using the trainable Weka segmentation plugin for ImageJ.^33^ The HAADF intensity was then used to generate a 3D-surface plot to visualize thickness variations. The SEEBIC images were used to record the location of the supported graphene and tinted blue in the 3D-renderings. Figure 6c shows an angled view of the composite rendering prior to heating. The overlay shows a top-down view. Figure 6f shows an angled view of the composite after the device failure. The overlay shows the top-down view.

It is interesting to note that in the HAADF image of the failed device, the location of the graphene bowtie can be clearly discerned. This is not due to contrast arising from the graphene layer itself since the graphene layer is not discernible in the original image. Rather, we observe differences in mass loss from the SiN_*x*_ substrate affected either by the presence of the graphene or a nonuniform thermal profile (or both). The region underneath the graphene appears brighter in the HAADF image than in the immediate surroundings, indicating that it is thicker. This is most likely due to the graphene mechanically preventing the substrate evaporation from one side (evaporation in other locations can occur from both the top and bottom sides of the thin SiN_*x*_ membrane). In addition, the edges of the ribbon appear darker than those of the surrounding exposed SiN_*x*_. It is possible for additional heating to occur at the edges. Two possible explanations for this are higher thermal resistance at graphene edges because of phonon scattering and phonon^53,54^ localization at the edges^55^ or increased electrical resistance due to edge disorders hindering the charge transport,^56^ which in turn may cause Joule heating or changes on the local Seebeck coefficient and therefore local Peltier heating/cooling.

A second example of these effects is shown in Figure 6g. A similar supported bowtie nanoribbon device was driven to failure and imaged. The HAADF intensity was converted to a thickness colormap as well as a displaced plane, where the extent of displacement is proportional to the HAADF intensity, overlaid. Intensity profiles were extracted from the HAADF image and are shown in the plot. The profiles were aligned using the edge of the hole as a reference point, and the intensity linearly scaled from 0 nm in vacuum to 20 nm in the SiN_*x*_ region. The profile from the ribbon edge exhibits a higher mass loss over a longer range than the other profiles. This is also visually apparent in the displacement map.

We have chosen the SiN_*x*_ membrane as a substrate because it is ideal for STEM experiments due to its ability to create apertures. The heat transport in these devices is limited mainly by the small thickness of the SiN_*x*_ membranes since the heat can flow mostly laterally within the membrane. As shown in the Supporting Information, the thermal boundary resistance between graphene and the substrate has little impact on the overall device heating. For device applications, a careful selection of substrates with better ability to dissipate heat is necessary. For example, graphene devices on a highly thermal conductive SiC substrate^57^ were found to break at much higher voltages than the ones on Si/SiO_2_/Si_3_N_4_ or Si/SiO_2_^58^ due to the better heat dissipation. However, a highly thermally conductive substrate does not always guarantee a better heat dissipation. Any thermal interface resistance between the different layers of a substrate can affect heat dissipation. For example, devices on low thermal conductive bulk quartz were found to sustain more current than those on highly conductive thin films of AlN or Al_2_O_3_ (deposited on Si/SiO_2_ wafers) due to the additional interface thermal resistances between the thin films and Si/SiO_2_ wafer, limiting the cross-plane heat dissipation,^27^ even if the in-plane heat dissipation within the thin layers is high.

The STEM experiments corroborate the observations from SThM and the FEA simulations regarding the Peltier shift in the temperature profile and indicate that this phenomenon exists up to device breakdown. Using direct observation of the graphene width coupled with current measurements enabled an estimate of the current density. We observed SiN_*x*_ substrate evaporation at current densities around 1.25 × 10^8^ Acm^–2^. The noncontact nature of STEM imaging allowed a detailed view of the breakdown process revealing locations of mass loss through HAADF imaging and correlation to the graphene locations through SEEBIC imaging.

The combined information from SThM, STEM, and FEA modeling provides detailed insight into the breakdown processes of graphene nanodevices operated in an ultrahigh current density regime. The role of interfaces and device geometry shown here suggests pathways toward controlling and confining the location of extreme operation to protect more delicate materials.

## Conclusions

We employed a series of *operando* SThM and STEM experiments coupled with FEA to examine the performance and failure modes of graphene nanodevices operated with current densities beyond the limits of material stability. We found that Peltier effects arising from the graphene geometry and interfaces with the surrounding materials play a crucial role in the failure modes. Specifically, narrowing down the graphene channel increases the electron scattering by the edges, which affects the local Seebeck coefficient, causing significant Peltier heating/cooling. Similarly, the substrate affects the graphene EMFP, and therefore, at any border of suspended/supported graphene, an important change of the local Seebeck coefficient and Peltier heating/cooling appears. In every case, we observed increased heat generation on the drain side of the device, which became the limiting factor and breakdown site. These results provide critical details about graphene nanodevice operation under extreme current density conditions, coupling direct observation with theoretical treatments. Future device design must include consideration of and may even leverage interfacial and geometrically designed Peltier effects into device functionality.

## Experimental Section

### Device Fabrication

In order to examine operational STEM and SThM compatible graphene-based devices, we used *in situ operando* device platforms described in a previous publication.^59^ Briefly, their fabrication was as follows: 1000 nm of thermal oxide was grown on 300 μm thick Si wafers. Twenty nm of Si-rich SiN_*x*_ was deposited using low-pressure chemical vapor deposition. EBL was used to pattern fine electrodes, and photolithography was used to pattern larger electrodes. Metalization was accomplished using e-beam evaporation (Cr 5 nm/Au 35 nm for fine electrodes and Au 95 nm for larger electrodes). Backside etching was used to define the electron transparent SiN_*x*_ windows beneath the device required for STEM examination. Reactive ion etching and anisotropic KOH etching at 80 °C were used. Apertures were milled in SiN_*x*_ using a gallium focused ion beam microscope. Wafer-scale graphene was grown and transferred to the device by Graphenea. The graphene was then patterned using EBL with a negative resist (AR-N 7500). O_2_ plasma etching was used to remove the unwanted graphene leaving devices between the electrodes. The chips were cleaned using *N*-methyl-2-pyrrolidone (NMP) followed by acetone and isopropyl alcohol to remove residual resist.

### Scanning Transmission Electron Microscopy

STEM imaging and characterization was performed using a Nion UltraSTEM 200. HAADF imaging was performed by using a nominal convergence angle of 30 mrad and an inner detector angle of 80 mrad. SEEBIC imaging was performed as described more fully elsewhere.^30^ Briefly, a Femto DLPCA 200 transimpedance amplifier operated at 10^11^ gain was used to record the current stripped from the sample by the electron beam. In this imaging mode, conductive regions connected to the transimpedance amplifier appear bright and disconnected or insulating regions appear dark. This allowed unambiguous visualization of the supported graphene layer on SiN_*x*_. EELS imaging was performed by using a Gatan Enfinium spectrometer. The nominal convergence and collection angles were 30 and 33 mrad, respectively. Core-loss quantification was performed using Quantifit.^60^ A Hartree–Slater model was used to fit the core loss peaks.

### Scanning Thermal Microscopy

SThM is an atomic force microscopy-based technique. A micromachined Si probe with a highly resistive region is close to the tip, which heats the tip when the current passes through it. The electrical resistance value when the tip is brought into contact with the sample depends on the tip–sample heat exchange and therefore the local thermal properties and the local temperature of the sample. By calibrating the electrical resistance with temperature and applying the appropriate model, we can get a thermal resistance^61−63^ map of the graphene devices (see also Supporting Information). For the thermovoltage maps, we use STGM, a technique we previously developed.^37^ It is an SThM-based technique in which the SThM tip acts as a local heater while raster scans the device, records the position-dependent open-circuit voltage drop on the source–drain, and constructs a thermovoltage map of the device. For the temperature maps, we used the technique developed by Menges *et al.*(64) In this technique, the SThM tip acts as a temperature sensor for the heated sample, and the temperature map is obtained from the difference of the measured heat flux of the Joule–Peltier heated (bias voltage is applied) and nonheated device (no bias voltage applied) (see Supporting Information for more details).

## Supporting Information Available

Details on SEM, SThM, analytical modeling, FEA, and examples of additional devices (PDF)
Movie of a device breakdown (AVI)

## Supplementary Material

nn3c12930_si_001.pdf

nn3c12930_si_002.avi
