# Supplementary material for: Thermoelectric Limitations of Graphene Nanodevices at Ultrahigh Current Densities
Source: ACS Nano. 2024 Apr 19;18(17):11153–64. doi: 10.1021/acsnano.3c12930 (PMC11064226; doi:10.1021/acsnano.3c12930)
Supplement: Supplementary file 1 — nn3c12930_si_001.pdf [file nn3c12930_si_001.pdf]

Supporting Information: Thermoelectric limitations of graphene nanodevices at ultra-high current densities

Charalambos Evangeli,[*,†,‡,⊥] Jacob Swett,[†,⊥] Jean Spiece,[¶,⊥] Edward McCann,[‡] Jasper Fried,[†] Achim Harzheim,[†] Andrew R. Lupini,[§] G. Andrew D. Briggs,[†] Pascal Gehring,[¶] Stephen Jesse,[§] Oleg V. Kolosov,[*,‡] Jan A. Mol,[‖] and Ondrej Dyck[§]

†*Department of Materials, University of Oxford, Oxford OX1 3PH, UK*

‡*Physics Department, Lancaster University, Lancaster LA1 4YW, United Kingdom*

¶*IMCN/NAPS, Université Catholique de Louvain (UCLouvain), 1348 Louvain-la-Neuve, Belgium*

§*Center for Nanophase Materials Sciences, Oak Ridge National Laboratory, Oak Ridge, TN*

‖*School of Physics and Astronomy, Queen Mary University of London, London E1 4NS, UK*

⊥*Equal Contribution*

E-mail: ch.evangeli@gmail.com; o.kolosov@lancaster.ac.uk

Contents

1 Scanning electron microscopy images of the chip **S-3**

2 Scanning Thermal Microscopy **S-4**

2.1 Thermal Resistance maps S-4
2.2 Temperature maps S-5
2.3 Peltier temperature maps when swapping source-drain S-8

3 Seebeck coefficient model **S-9**
3.1 Seebeck coefficient with a sharp spatial dependence (bowtie supported device) S-9
3.2 Gaussian temperature spot S-10
3.3 Seebeck coefficient with three different values (bowtie suspended device) . . S-10

4 Transmission Electron Microscopy **S-11**
4.1 Electron Energy Loss Spectroscopy S-11
4.2 SEEBIC Composite S-12

5 Finite Element Analysis **S-14**
5.1 Procedure S-14
5.2 Ribbon supported S-17
5.3 Temperature maps S-18
5.4 Thermal conductivity-dependent temperature S-19
5.5 Current density-dependent temperatures S-21
5.6 Impact of the thermal boundary resistance between graphene and silicon nitride S-23
5.7 Effect of Swapping drain and source on Peltier heating/cooling direction . . S-24
5.8 Impact of aperture direction and dimension S-24

6 Ballistic heat transport **S-26**

7 Additional Data **S-28**
7.1 Resistance evolution S-28
7.2 Additional devices S-29

References **S-30**

1 Scanning electron microscopy images of the chip

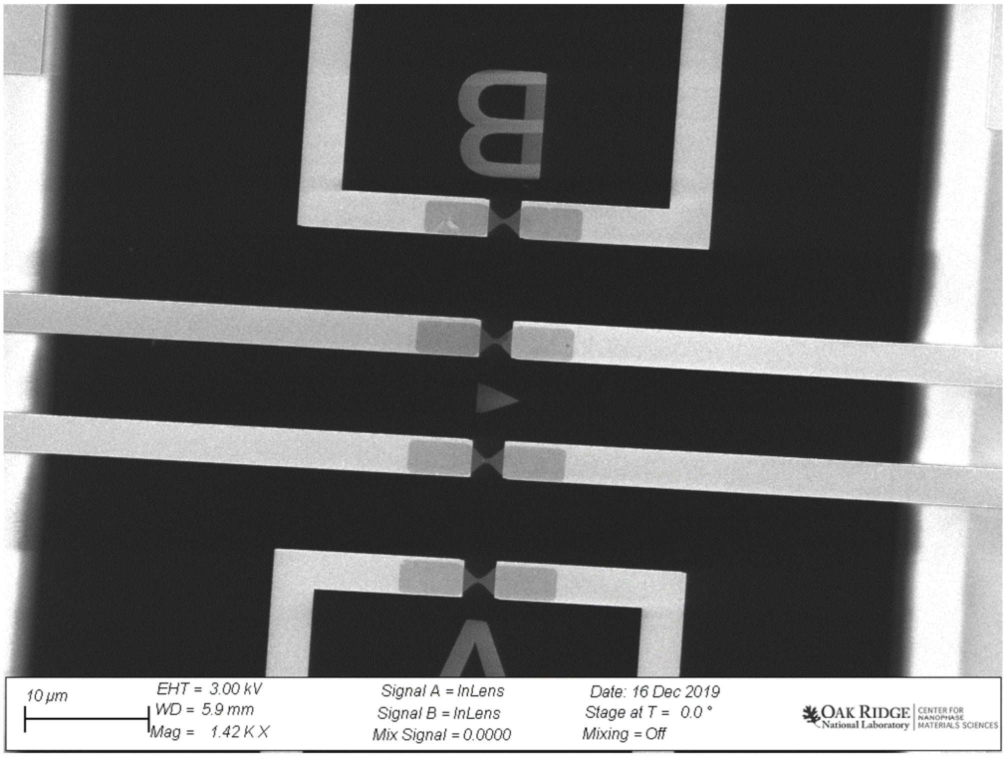

Figure S1: Scanning electron microscopy image of a typical chip with 4 graphene bowtie devices on SiN_x membrane.

2 Scanning Thermal Microscopy

2.1 Thermal Resistance maps

When the SThM probe is brought in- and out- of contact to a non-biased graphene device the normalized temperature change between out- and in- contact is given by the formula:[S1]

$$\frac{\Delta T_{\rm nc} - \Delta T_{\rm c}}{\Delta T_{\rm nc}} = \frac{V_{\rm nc} - V_{\rm c}}{V_{\rm nc}} = \frac{1}{R_{\rm p} + R}(R_{\rm p} + \frac{T_{\rm M} - T_{\rm S}}{Q_{\rm h}}), \quad (1)$$

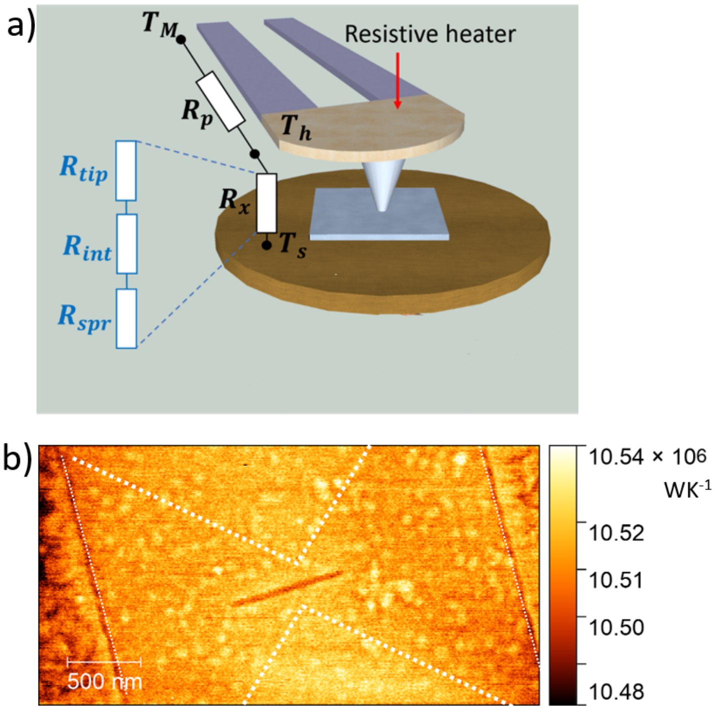

Figure S2: a) Thermal circuit of SThM tip and sample. (b) Thermal Resistance map for the device presented in Figure 2(a-d) in the main text

where $T_{\rm M}$ and $T_{\rm S}$ the macroscopic temperature of the microscope and the sample respectively which are being monitored by Pt-resistive elements, $\Delta T_{\rm nc} = T_{\rm nc} - T_{\rm M}$ and $\Delta T_{\rm c} = T_{\rm c} - T_{\rm M}$ are the excess temperatures of the probe when out- ($T_{\rm nc}$) and in- ($T_{\rm c}$) contact respectively, with respect to the microscope temperature, $V_{\rm nc}$ and $V_{\rm c}$ is the SThM signal for out- and in- contact respectively, $R_{\rm p}$ is the thermal resistance of the SThM probe, $R_{\rm X}$ is

the thermal contact resistance of the tip-sample contact and Q_{h} is the heat generated in the probe heater. Note, that $R_{\mathrm{X}} = R_{\mathrm{tip}} + R_{\mathrm{int}} + R_{\mathrm{spr}}$, where R_{tip},R_{int},R_{spr} are the tip, sample tip interface, and sample spreading resistances, respectively.

T_{M} and T_{S} are monitored during the experiment and Q_{h} and R_{p} are known through the probe calibration as detailed elsewhere.[S1,S2] Briefly, the power applied to the probe heater gives rise to an excess temperature,ΔT), with respect the microscope temperature, T_{M}, that relates to the SThM output voltage by a calibration factor b,$\Delta T_{\mathrm{tip}} = bV$. V_{nc} does not depend on the position of the probe, therefore, by replacing V_{c} with the SThM signal that is obtained during scanning the sample we can quantify the nanoscale SThM map in terms of thermal contact resistance R_{X}. A thermal resistance map of the device presented in Figure 3 (a-d) in the main text is shown in figure S2(b). Note that the suspended graphene is more thermally conductive than the supported.

2.2 Temperature maps

The temperature maps were obtained with the technique developed by Menges et al.[S3] In this technique the temperature map is obtained from the difference between the images of the device with and without heat flux due to Peltier/Joule heating, therefore with and without applying any bias voltage. More specifically, after after obtaining the thermal Resistance images without applying, $R_{\mathrm{X'}}$, and by applying, R_{X}, bias voltage we can extract the absolute temperature map with the following formula:

$$\Delta T_{\mathrm{sample}} = \Delta T_{\mathrm{tip}} \left(1 - \frac{R_{\mathrm{X}}}{R_{\mathrm{X'}}}\right)$$

(2)

with ΔT_{tip} being the excess temperature of the probe as obtained from the probe cali-

bration.

In the following temperature maps of device 1 and 2 (bowtie shaped graphene on SiN membrane with an aperture along the device) are presented. Note, that the 2 devices were measured with different tips. A direct comparison of the absolute temperatures between the two devices is misleading due to uncertainties added by the different probes used. More specifically, for device 2, a probe with larger contact area comparable to the slit width was used, resulting less sensitivity on the suspended part temperature. When the tip pass through the suspended part, due to its size, it feels an average temperature of the suspended and supported part. Therefore, the temperature rise in the suspended part is less pronounced. This can be seen also in the 2D temperature map of the Fig.3e in the manuscript.

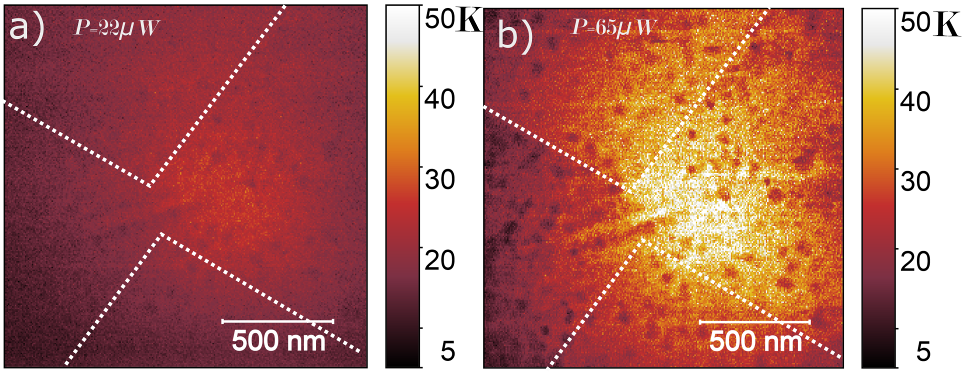

Figure S3: Device 2: (a,b) Temperature maps for $P = 22\mu W$ (a) and $P = 65\mu$ (b). By increasing the Power applied on the devices the temperature rises in a larger area. The temperature rise is asymmetric towards the drain side of the device.

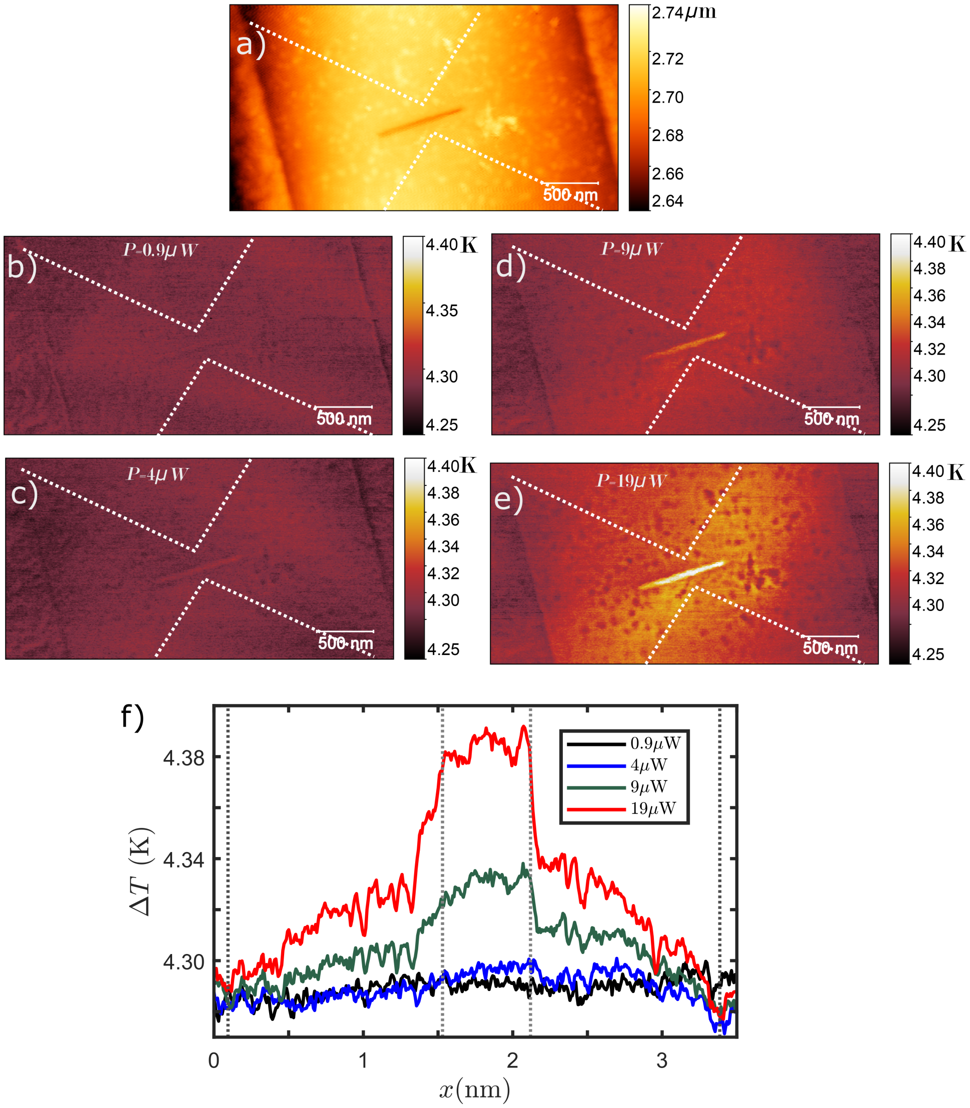

Figure S4: Device 1: (a) Topography map.(b-e) Temperature maps for $P = 0.9\mu$W (b), $P = 4\mu$W (c), $P = 9\mu$W (d),$P = 19\mu$W (e). (f) Temperature profiles along the slit for the different Power applied on the device. Dotted vertical lines indicate the gold contacts and the SiN slit's edges. The temperature at the gold contacts remains almost stable, indicating that gold acts as a heat sink, for low bias currents.

2.3 Peltier temperature maps when swapping source-drain

Figure S5 shows Peltier temperature maps of a bowtie graphene device on SiO_2 when swapping the source and drain. The maps were measured with the technique described in our previous work.[S4] As can be seen by swapping the source and drain the Peltier heating and cooling are also reversed.

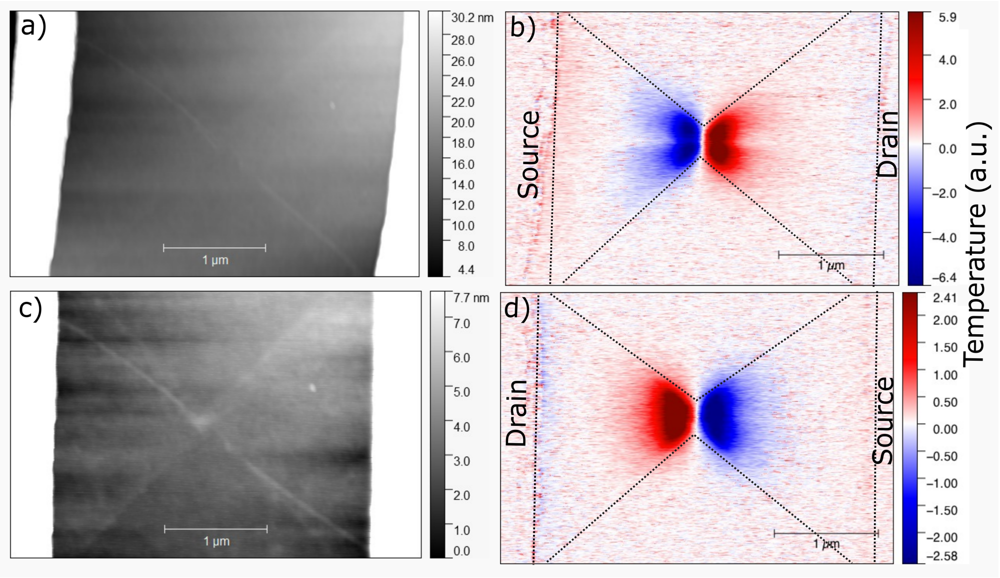

Figure S5: Peltier Temperature (b,d) and topography maps (a,c) of graphene bowtie device on SiO_2 when swapping the source and drain. (b,c) Adapted by permission from American Chemical Society, copyright 2018, Ref.[S4]

3 Seebeck coefficient model

3.1 Seebeck coefficient with a sharp spatial dependence (bowtie supported device)

The thermovoltage V_{th} is given by

$$V_{\text{th}} = -\int_{x_L}^{x_R} S(x)\frac{dT(x)}{dx}dx, \tag{3}$$

where $S(x)$ is the Seebeck coefficient. We mimic the form of $S(x)$ in Ref.[S4] with

$$S(x) = S_0 - L_S S_0 \delta(x - x_0), \tag{4}$$

where S_0 (seebeck coefficient of graphene) and L_S (width of the graphene constriction) are constants (independent of x). This is essentially valid when the spatial dependence of $S(x)$ is a lot faster than that of $T(x)$. With this, the thermovoltage is

$$\begin{aligned} V_{\text{th}} &= -\int_{x_L}^{x_R} S_0\frac{dT}{dx}dx + \int_{x_L}^{x_R} S_0 L_S \delta(x - x_0)\frac{dT}{dx}dx \\ &= -S_0\int_{x_L}^{x_R} \frac{dT}{dx}dx + S_0 L_S\int_{x_L}^{x_R} \delta(x - x_0)\frac{dT}{dx}dx \\ &= -S_0[T(x)]_{x_L}^{x_R} + S_0 L_S \frac{dT}{dx}\bigg|_{x_0} \\ &= -S_0\left[T(x_R) - T(x_L)\right] + S_0 L_S \frac{dT}{dx}\bigg|_{x_0} \\ &= S_0 L_S \frac{dT}{dx}\bigg|_{x_0}, \end{aligned} \tag{5}$$

where we assume that the boundary term vanishes due to $T(x_R) = T(x_L)$. We have in mind $x_0 = 0$, so that

$$V_{\text{th}} = S_0 L_S \frac{dT}{dx}\bigg|_{0}. \tag{6}$$

Note, x_{L}, x_{R} is the position of the left and right electrodes of the device,

3.2 Gaussian temperature spot

Before solving the heat equation, let's assume that there is a localised Gaussian temperature spot centred on $x = x_t$:

$$T(x) = T_0 + (T_t - T_0)e^{-(x-x_t)^2/(2\sigma^2)}, \tag{7}$$

where T_t (excess temperature of the tip) and σ (standard deviation of the Gaussian temperature hot spot) are constants and T_0 is the room temperature. Thus,

$$\frac{dT(x)}{dx} = -\frac{(T_t - T_0)(x - x_t)}{\sigma^2} e^{-(x-x_t)^2/(2\sigma^2)}, \tag{8}$$

and with Eq. (6):

$$V_{\mathrm{th}} = \frac{S_0 L_s (T_t - T_0) x_t}{\sigma^2} e^{-x_t^2/(2\sigma^2)}. \tag{9}$$

This assumes that $L_s \ll \sqrt{\sigma}$.

3.3 Seebeck coefficient with three different values (bowtie suspended device)

To mimic a dependence of $S(x)$ due to a bowtie constriction (giving a value of $S(x) = 0$ over a width of length L_s about $x = 0$) and a dependence due to a slit (giving a value of

$S(x) = S_1$ over a width of length s about $x = 0$), write

$$S(x) = \begin{cases} S_0 & \text{for } |x| > L_s/2 \\ S_1 & \text{for } s/2 < |x| < L_s/2 \\ 0 & \text{for } |x| < s/2 \end{cases} \tag{10}$$

where $L_s < s$. Then (3) gives

$$\begin{aligned} V_{\text{th}} &= S_1 \left[T(s/2) - T(-s/2)\right] \\ &\quad + (S_0 - S_1)\left[T(L_s/2) - T(-L_s/2)\right], \end{aligned} \tag{11}$$

where we assume that the boundary term vanishes. With the Gaussian temperature (7):

$$\begin{aligned} V_{\text{th}} &= (S_0 - S_1)(T_t - T_0)\left[e^{-\frac{(x_t - L_s/2)^2}{2\sigma^2}} - e^{-\frac{(x_t + L_s/2)^2}{2\sigma^2}}\right] \\ &\quad + S_1(T_t - T_0)\left[e^{-\frac{(x_t - s/2)^2}{2\sigma^2}} - e^{-\frac{(x_t + s/2)^2}{2\sigma^2}}\right]. \end{aligned} \tag{12}$$

The above equation is the one used to fit the experimental thermovoltage curve of figure 3 of the main text.

4 Transmission Electron Microscopy

4.1 Electron Energy Loss Spectroscopy

In Figure S6 we examine the composition of the device shown in Figure 2 (e-h) of main text using core loss electron energy loss spectroscopy (EELS) analysis. We captured an EELS image across the burned area after device failure and quantified the relative composition using the Si, C, and N core loss edges. An example background-subtracted spectrum is shown overlaid on Figure S6a taken from the region indicated by the colored box overlay.

Figure S6c-e show the relative concentration of Si, C, and N acquired using Hartree-Slater model fitting. The SiN_x composition was then calculated as a function of position and is shown in Figure S6b. A mean value of 1.05 was found in the pristine region (calculated using the bottom five rows of pixels). Regions exhibiting significant mass loss show an increase in N concentration which suggests a preferential loss of Si during failure. This is somewhat unexpected given that N is the lighter element, however this may be indicative of a transition to Si_3N_4 which is consisten with a value of x=1.33.

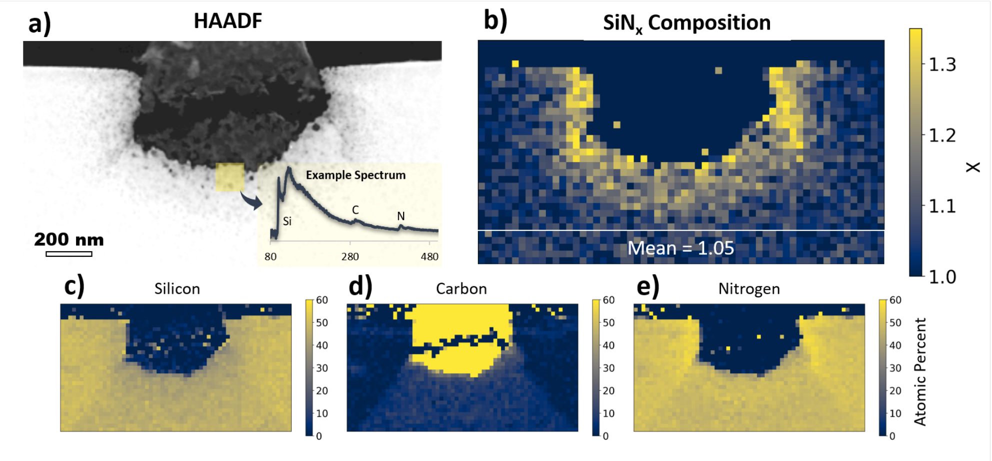

Figure S6: Summary of an EELS spectrum image acquired on the same device shown in Figure 5 of main text. The Si L_{23}, C K, and N L_{23} edges were captured. a) shows a HAADF image of the location. Inset is an example background-subtracted spectrum from the indicated region. c)-e) show atomic concentration maps for Si, C, and N respectively. b) shows a map of the N and Si composition. We observe a pronounced increase in N at the edge of the SiN_x.

4.2 SEEBIC Composite

HAADF and SEEBIC image channels were recorded in parallel ensuring a one-to-one positional correspondence. Figure 5 of the main text features HAADF/SEEBIC composite images. Here, we describe the proceedure used to create the composite images. The SEEBIC signal was first corrected for periodic interference using a procedure similar to that used for dark correction on pixelated detectors. An example corrected SEEBIC image is shown in Figure S7b. Overlaying this SEEBIC image on the HAADF results in unnecessary obfus-

cation of the HAADF image. Instead we segmented the SEEBIC image into conductive and non-conductive (i.e. bright and dark) regions using the trainable weka segmentation plugin for Imagej.[S5] These regions are shown colored in Figure S7c. The non-conductive regions labeled "Discard" were removed and the remaining regions were overlaid on the HAADF image as shown in Figure S7d.

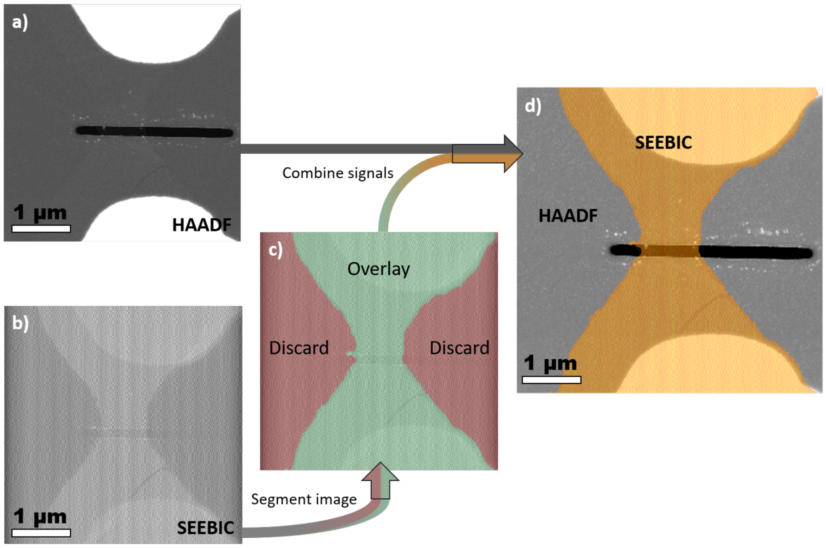

Figure S7: Summary of the procedure used to generate HAADF/SEEBIC composite images . a) HAADF and b) SEEBIC images. c) segmented regions of the SEEBIC image. Areas labeled "Discard" were not included in the overlay. d) final HAADF/SEEBIC composite image.

5 Finite Element Analysis

5.1 Procedure

To calculate the Joule heating and Peltier temperature maps, we turned to Finite Element Analysis (FEA) using COMSOL MULTIPHYSICS©. Realistic 3D models included heat transfer in solid, electrical current and multiphysics effects, e.g. thermoelectric effects. The device geometry was known from manufacturing. It consists in a graphene layer resting on a silicon nitride 20 nm thick membrane. The model assumes that the edge of the membrane are at equilibrium with a thermal bath. The graphene layer geometry is varied but typically has lateral dimensions of 3μm. The silicon nitride membrane is modelled as a square of 30μm. The graphene thickness is chosen to be 1 nm. The device (graphene layer and electrodes) is resting on a silicon nitride membrane and therefore the only connections to the thermal bath are *via* the edges of the membrane. In addition, we assumed perfect coupling between the graphene and the silicon nitride. However, the impact of a thermal boundary resistance between the grpahene layer and the silicon nitride membrane is studied later in section 5.6. The electrodes material was set to graphene instead of gold. This was to avoid high thermoelectric effects between the electrodes and the graphene layer that would hide our zone of interest in the middle of the device. Although, for one of the geometries, we show the results with gold electrodes in figure S9. Graphene was modeled as a uniform layer with given thermal and electrical conductivities, given in the table S1 and the Seebeck coefficient was assumed uniform perpendicularly to the device axis. The Seebeck coefficient modeled in the main text is set for the graphene Seebeck coefficient and is thus position dependent.

The main equations solved for this system are:

$$\rho C_p \frac{\partial T}{\partial t} + \nabla(-k\nabla T + P\mathbf{J}) = Q \tag{13}$$

$$\mathbf{J} = -\sigma(\nabla V + S\nabla T) \tag{14}$$

$$\tag{15}$$

where ρ, C_P, k, σ are materials properties of density, heat capacity and thermal and electrical conductivities, given in tables S1 and S2, respectively. The graphene thermal and electrical conductivities in the supported case were chosen to reproduce the temperature distribution measured experimentally. For the suspended case, unless specified otherwise, we used the experimental thermal conductivity obtained by fitting the suspended region with model described in the main text. $T, Q, \mathbf{J}, V$ are temperature, heat density, current density and potential, respectively. S and P are the Seebeck and Peltier coefficients with $P = ST$. The first equation is the heat flux equation where a $P\mathbf{J}$ contribution is added to take into account thermoelectric effects while the second adds the therm $-\sigma S \nabla T$ to the current density.

Table S1: Graphene parameters used in simulations

Density	1950 kgm^{-3}
Heat capacity	710 $\text{Jkg}^{-1}\text{K}^{-1}$
Electrical conductivity	5×10^5 Sm^{-1}
Thermal conductivity supported	120 $\text{Wm}^{-1}\text{K}^{-1}$

Table S2: Silicon nitride parameters used in simulations

Density	3100 kgm^{-3}
Heat capacity	700 $\text{Jkg}^{-1}\text{K}^{-1}$
Electrical conductivity	0 Sm^{-1}
Thermal conductivity	2.5 $\text{Wm}^{-1}\text{K}^{-1}$

We proceeded as follows. One electrode of the device is grounded while on the other electrode we apply a DC current. Then, we compute the resulting temperature field produced in the device by the thermoelectric effects. Then we change the polarity of the DC current. The obtained temperature fields include both Peltier and Joule effects. To separate them, we extract modeled temperature profiles from both simulations (T_{DC^+} and T_{DC^-}) and define the Peltier and Joule temperatures as

$$T_{Peltier}(x) = \frac{T_{DC^+}(x) - T_{DC^-}(x)}{2} \quad (16)$$

$$T_{Joule}(x) = \frac{T_{DC^+}(x) + T_{DC^-}(x)}{2} \quad (17)$$

We computed Peltier and Joule effects using Finite Element Analysis on three device geometries (see Fig. S8): a ribbon with a slit in the middle, a supported bowtie and a bowtie with a slit in the middle. For each geometry, the position dependent Seebeck coefficient was given.

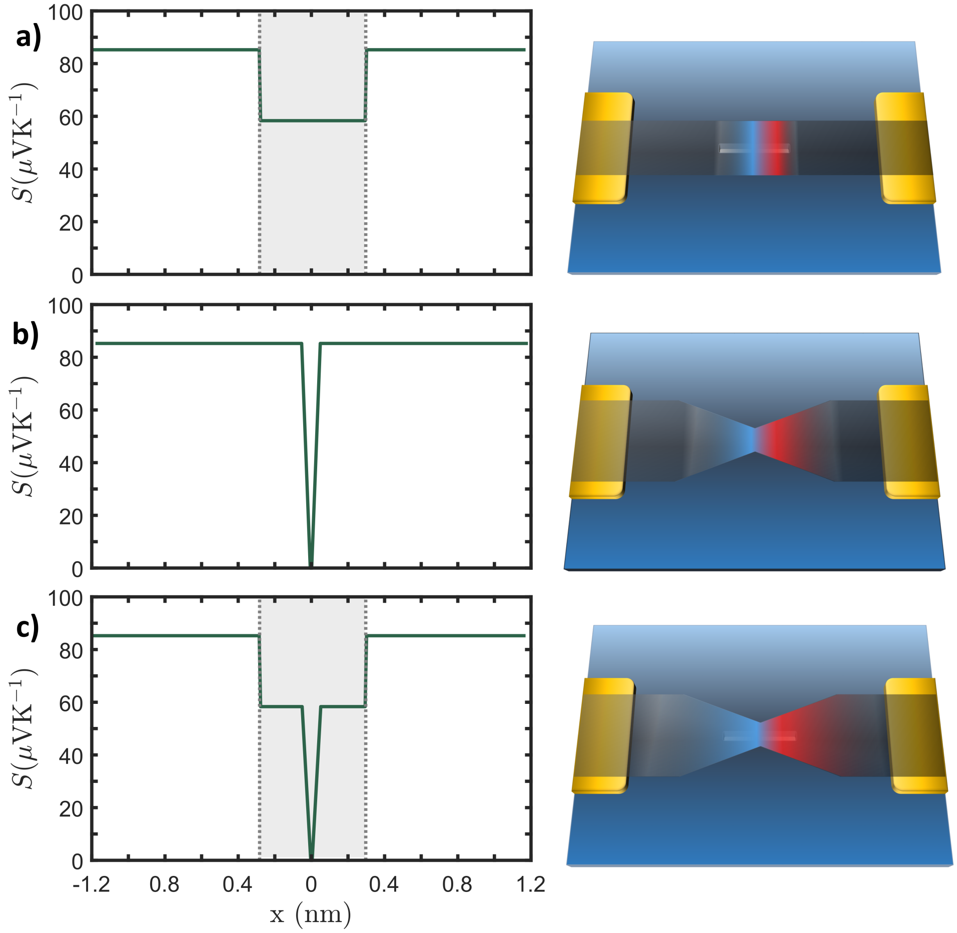

Figure S8: (a-c) Seebeck coefficient profiles used as input for the FEA calculations for the squared ribbon with suspended part (a), the bowtie (b), and the bowtie with suspended part (c).

The profile in Figure S8c was extracted by fitting the phenomenological model on the experimental data. The absolute values of Seebeck coefficient found with this fitting were used to extract the other two profiles. More specifically, the profile in Figure S8b is a mimic of the form by Harzheim et.al.[S4] and in Figure S8c by assuming two different values of Seebeck coefficient for the suspended and supported part, respectively.

5.2 Ribbon supported

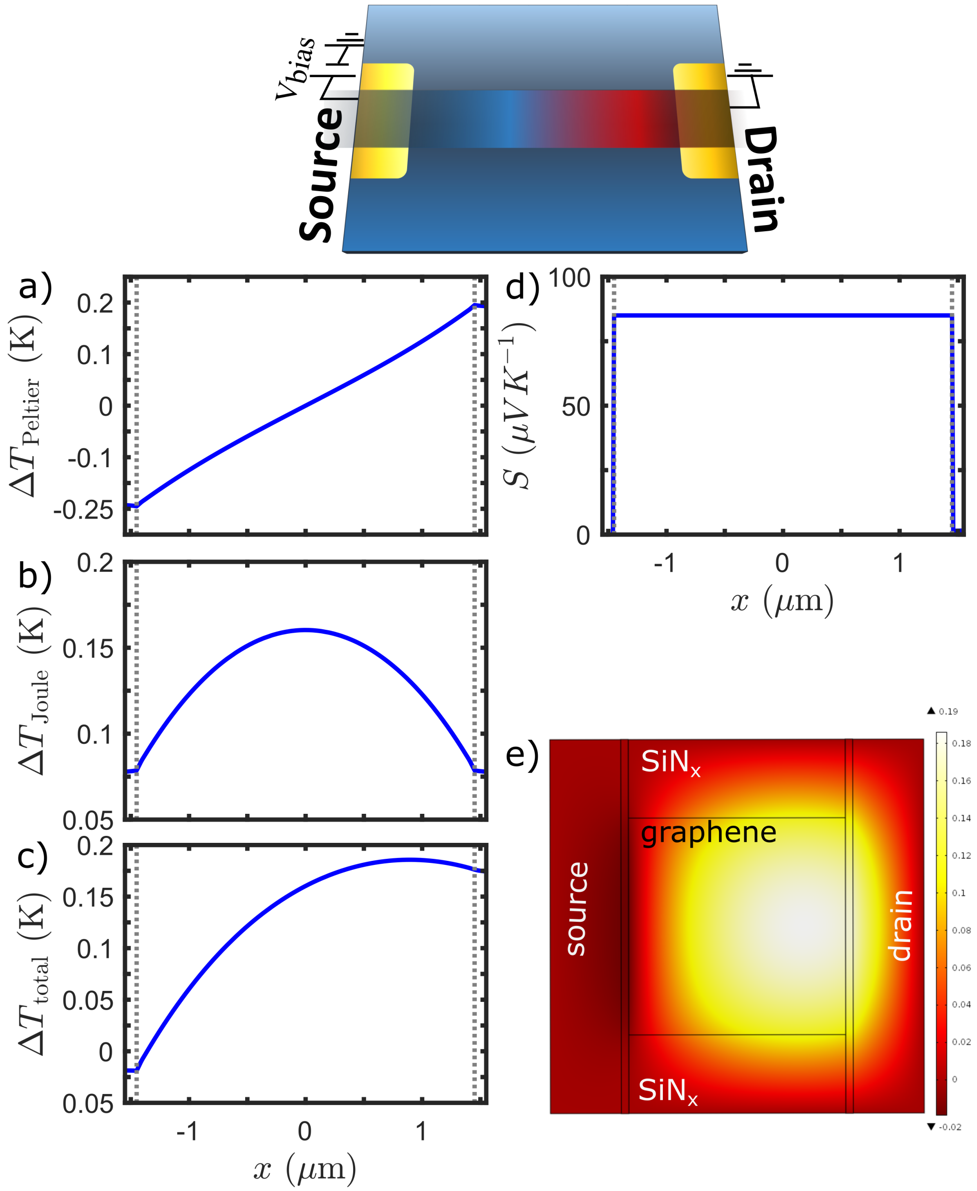

Figure S9: (a-c) Temperature profiles for squared graphene ribbon on SiN_x membrane. Peltier heating/cooling (a), Joule heating (b) and total (c) temperature. (d) Seebeck coefficient profile used as input in FEA calculations. Also, thermal conductivity value of 120 $Wm^{-1}K^{-1}$ for graphene was used. (e) Temperature map as acquired from FEA calculations for the sample device.

The Joule heating is very small while Peltier heating/cooling appears at the graphene/gold contact due to the different Seebeck coefficients of graphene and gold. The overall tempera-

ture increases at the drain side of the device.

5.3 Temperature maps

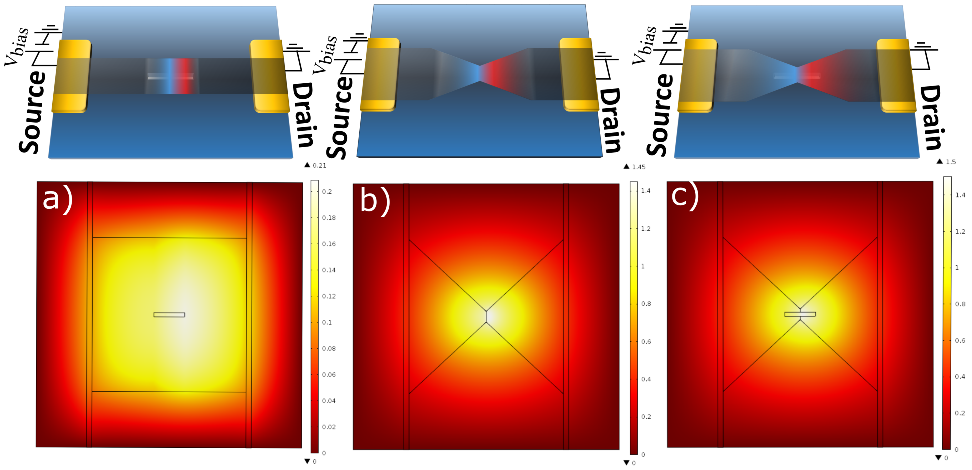

Figure S10: (a-c) Temperature maps as observed by FEA of a squared graphene ribbon on SiN_x membrane with aperture (a), of a bowtie device on SiN_x membrane without (b) and with (c) aperture.

Using the Seebeck coefficients as represented in Fig.S8, we computed the temperature maps of the three geometries. These temperature maps are used to extract temperature, Peltier and Joule heating profiles shown in the main text.

5.4 Thermal conductivity-dependent temperature

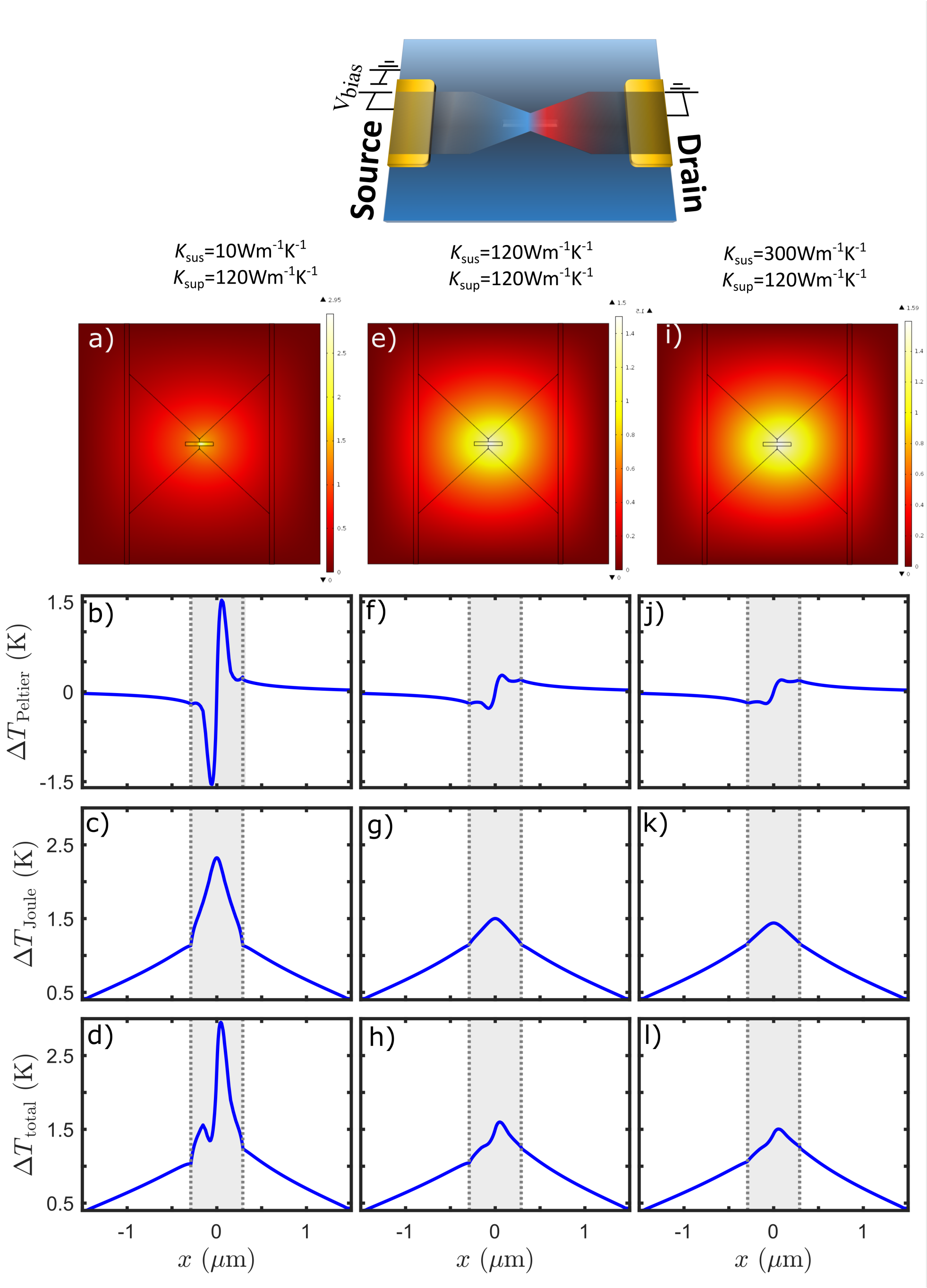

Figure S11: Temperature maps and profiles for bowtie graphene on SiN with aperture. Temperature maps (a,e,i) Peltier heating/cooling (b,f,j), Joule heating (c,g,k) and total (d,h,l) temperature. For the supported graphene, thermal conductivity value of 120 Wm^{-1}K^{-1} was used as input in the FEA calculations, while 3 different values were used for the suspended part: 10 Wm^{-1}K^{-1} (a,b,c), 120 Wm^{-1}K^{-1} (d,e,f) and 300 Wm^{-1}K^{-1} (g,h,i). Note that, the grey shaded region is the suspended part and the borders of the SiN$_x$ window are shown with dotted grey lines.

In order to investigate the effect of the suspended graphene on the overall temperature map, we used different thermal conductivities for the suspended part. This study might not be physically relevant and is mostly to investigate the trend created by changing the suspensed thermal conductivity k_{sus}.

Three different values where used for k_{sus}: $k = 10$ $Wm^{-1}K^{-1}$, $k = 120$ $Wm^{-1}K^{-1}$ as for the supported part and $k = 300$ $Wm^{-1}K^{-1}$. The results are shown on Fig. S11 for the bowtie geometry and on Fig. S12 for the ribbon geometry.

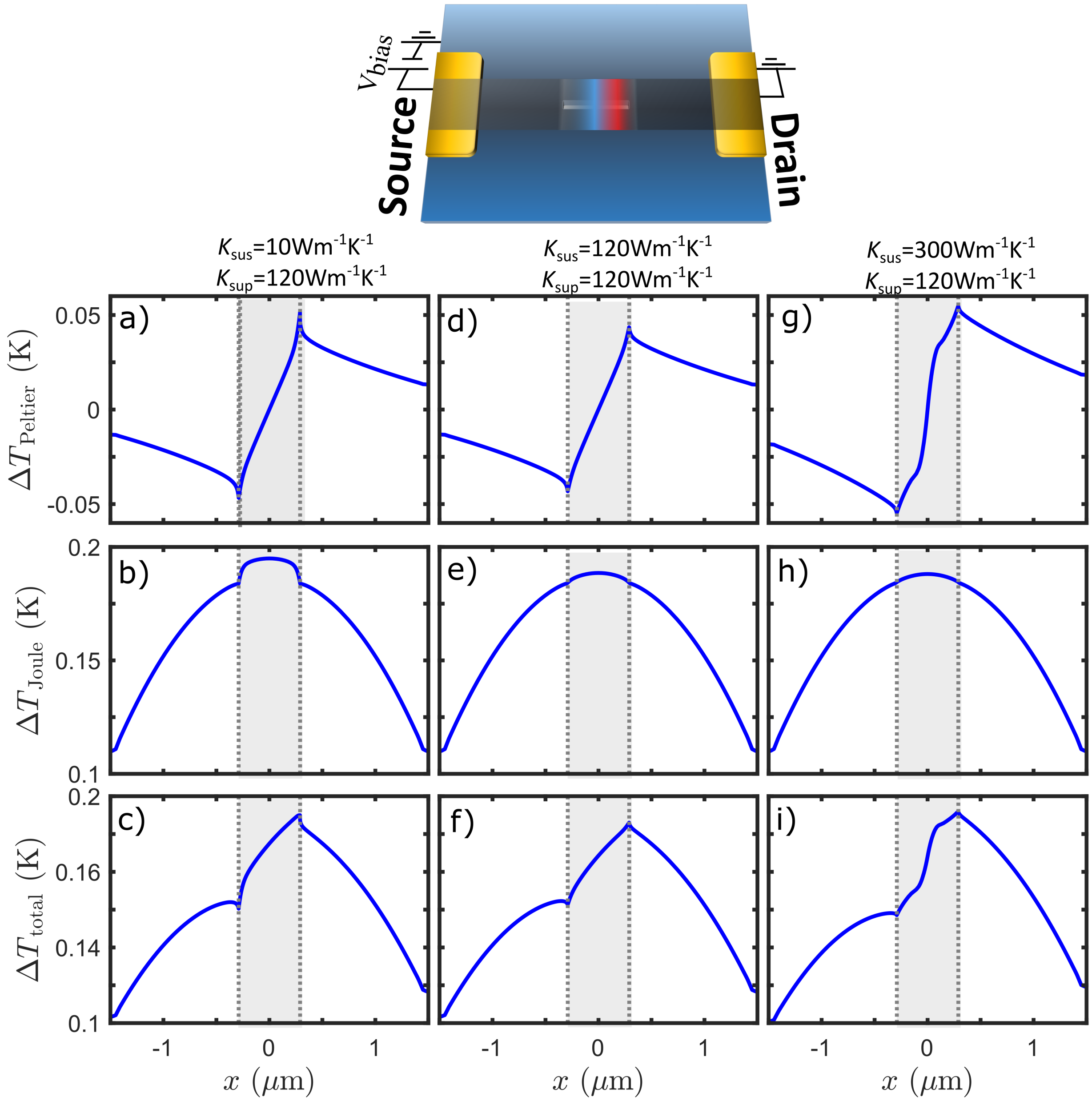

Figure S12: Temperature profiles for squared ribbon graphene on SiN with aperture. Peltier heating/cooling (a,d,g), Joule heating (b,e,h) and total (c,f,i) temperature. For the supported graphene, thermal conductivity value of $120Wm^{-1}K^{-1}$ was used as input in the FEA calculations, while 3 different values were used for the supported part: $10Wm^{-1}K^{-1}$ (a,b,c), $120Wm^{-1}K^{-1}$ (d,e,f) and $300Wm^{-1}K^{-1}$ (g,h,i). Note that, the grey shaded region is the suspended part and the borders of the SiN_x window are shown with dotted grey lines.

5.5 Current density-dependent temperatures

We computed Peltier and Joule effects using several input currents on the bowtie with a slit geometry. Currents were ranging from 5 to 55 μA (see Fig. S13). Both effects are increasing

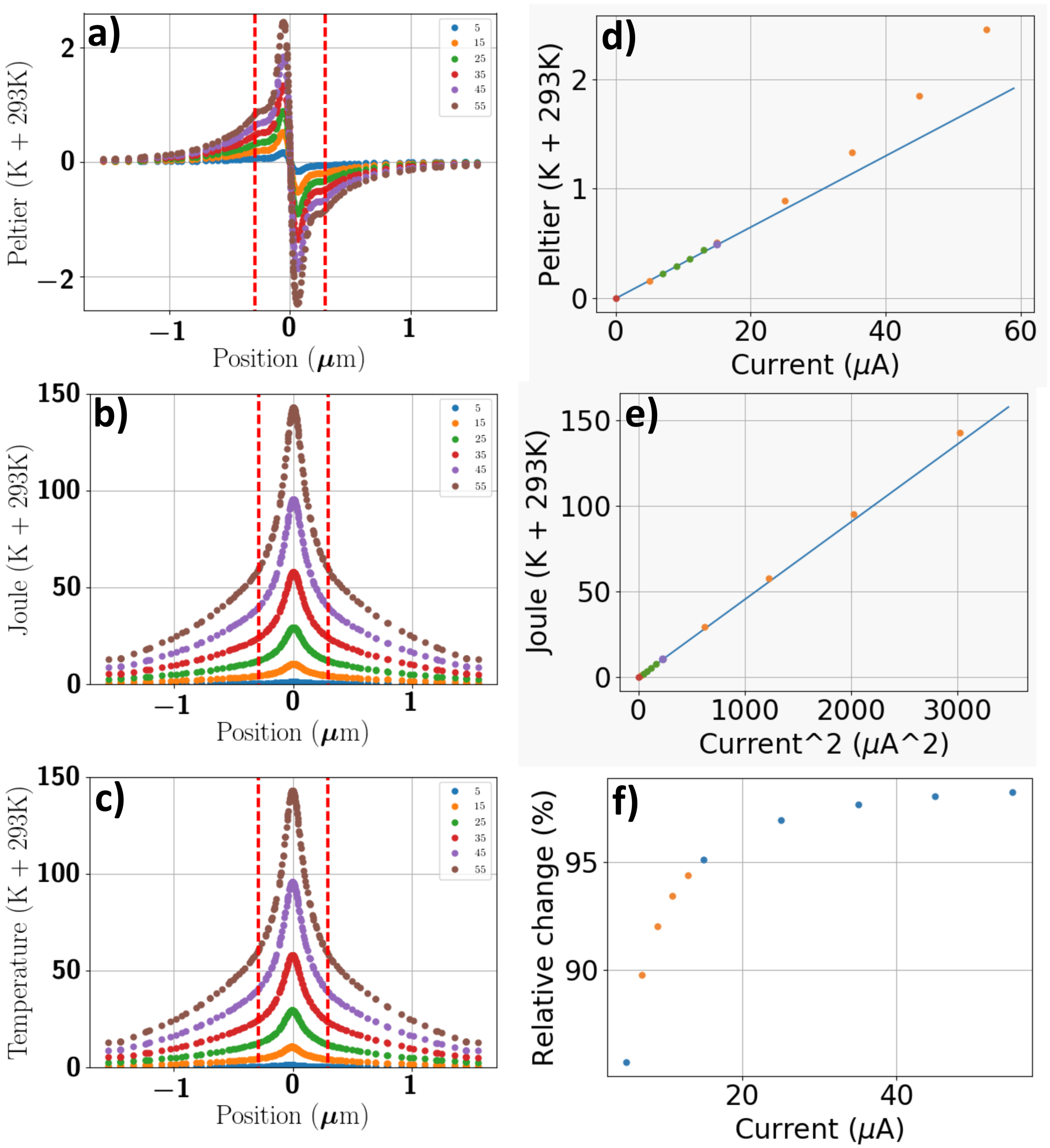

Figure S13: (a-c) Peltier (a), Joule (b) and total (c) temperature profiles for different bias currents. (d,e) Maximum Peltier (d) and Joule temperatures (e) of the device vs bias current. (f) Relative change of the Peltier and Joule effects.

with current as expected. Indeed, the Peltier effect is supposed to be linear with current while Joule effect is supposed to scale as current square.

At low current, the modeled effects are linear, as expected. However, we obtained a non

linear behaviour of both effects at high current. We believe that this might arise from boundary conditions. As the device size is not infinite, the temperature set as boundary condition on the edges of the device (room temperature) influences the device temperature. This is especially true at high current as the heat generated in the device creates a temperature field with a higher gradient due to the boundary condition.

5.6 Impact of the thermal boundary resistance between graphene and silicon nitride

In this section, we investigate the impact of a thermal boundary resistance (TBR) between graphene and silicon nitride on the simulated heating of a bowtie device. We should first highlight that direct comparison between experimental results and finite element modelling is not straightforward. Using the materials parameters listed in tables S1 and S2, we set the current as in our experiment (14 μA). However, therer are still a number of modelling parameters that have to be chosen arbitrarily making the model appropriate for qualitative understanding but not for quantitative comparison. With these parameters, we obtained a resulting temperature rise around 50.4 K which is comparable to the experimental value for the given current. We then increase the TBR over two orders of magnitude with values from 1×10^{-9} to 1×10^{-7} m^2KW^{-1}. Results are shown on figure S14. As it can be observed, the magnitude of the extra heating due to the TBR is small even with high TBR values. This could be expected as the heat transport in limited by the SiNx membrane thickness (20nm). Since the heat can only flow laterally within the SiNx membrane, the extra TBR does not affect much the overall thermal resistance experienced by the graphene device.

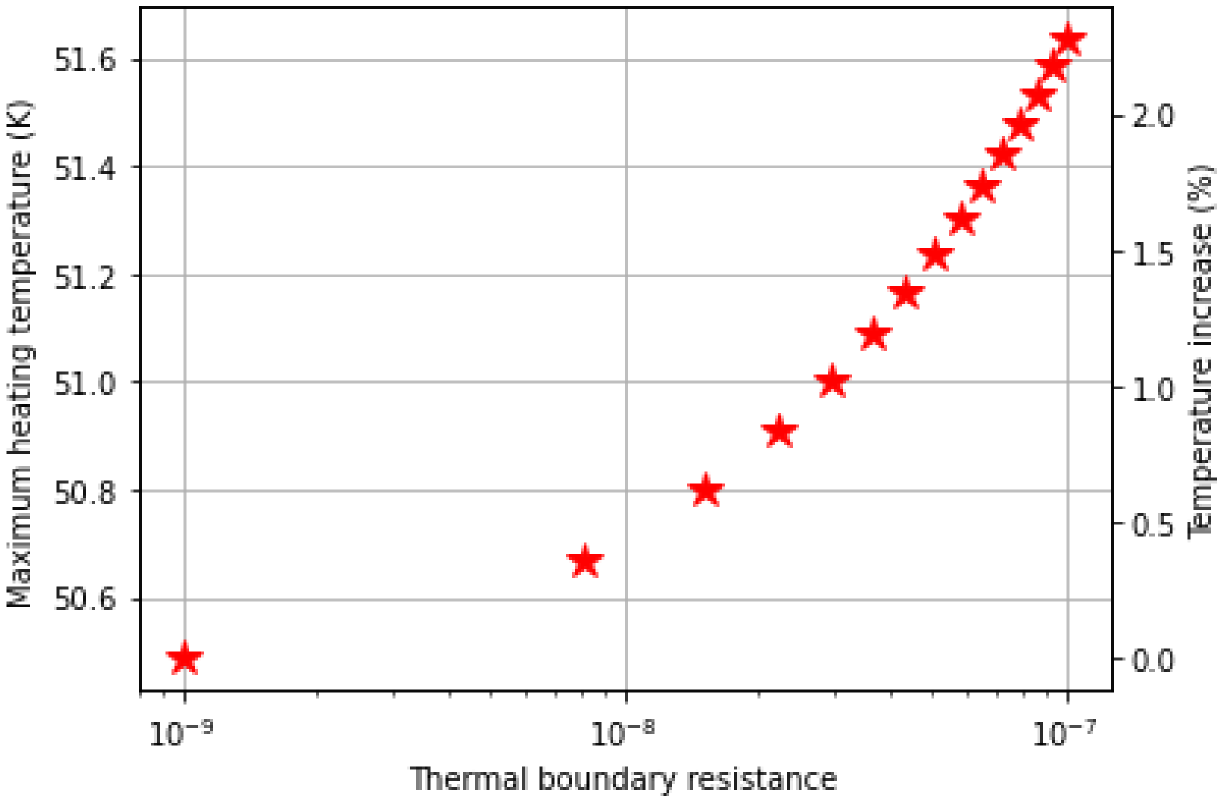

Figure S14: Maximum temperature and temeprature increase as a function of thermal boundary resistance between graphene and silicon nitride

5.7 Effect of Swapping drain and source on Peltier heating/cooling direction

To confirm the effect of swapping drain and source on the Peltier, we performed some simulations. As shown on figure S15, when the drain and source are swapped, the Peltier heating and cooling are also reversed.

5.8 Impact of aperture direction and dimension

Here, we investigate if the aperture direction could impact the direction of the Peltier heat. We investigated 2 directions: (i) along the channel direction as in SThM experiments and (ii) perpendicular to the channel as in the STEM experiments. Results are displayed on figure S16. As it can be observed, the direction doesn't impact the Peltier heat direction.

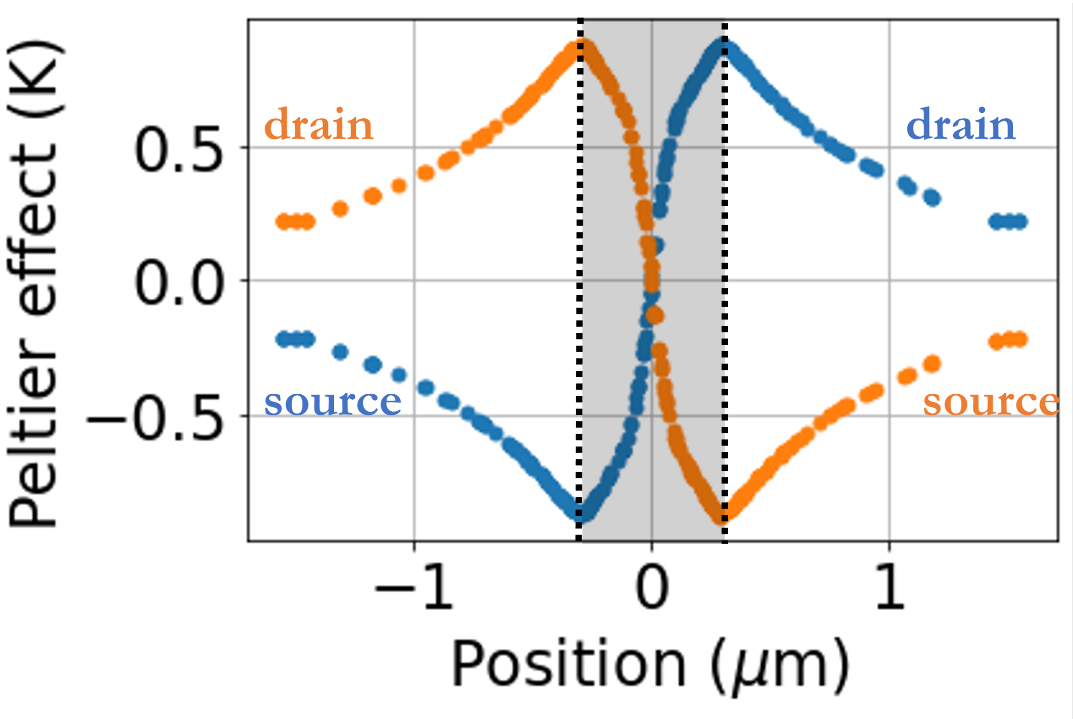

Figure S15: Simulation of the Peltier temperature when the drain and source and swapped. The suspended part is indicated by the shaded region.

The Peltier effect is slightly reduced for the aperture perpendicular to the channel.

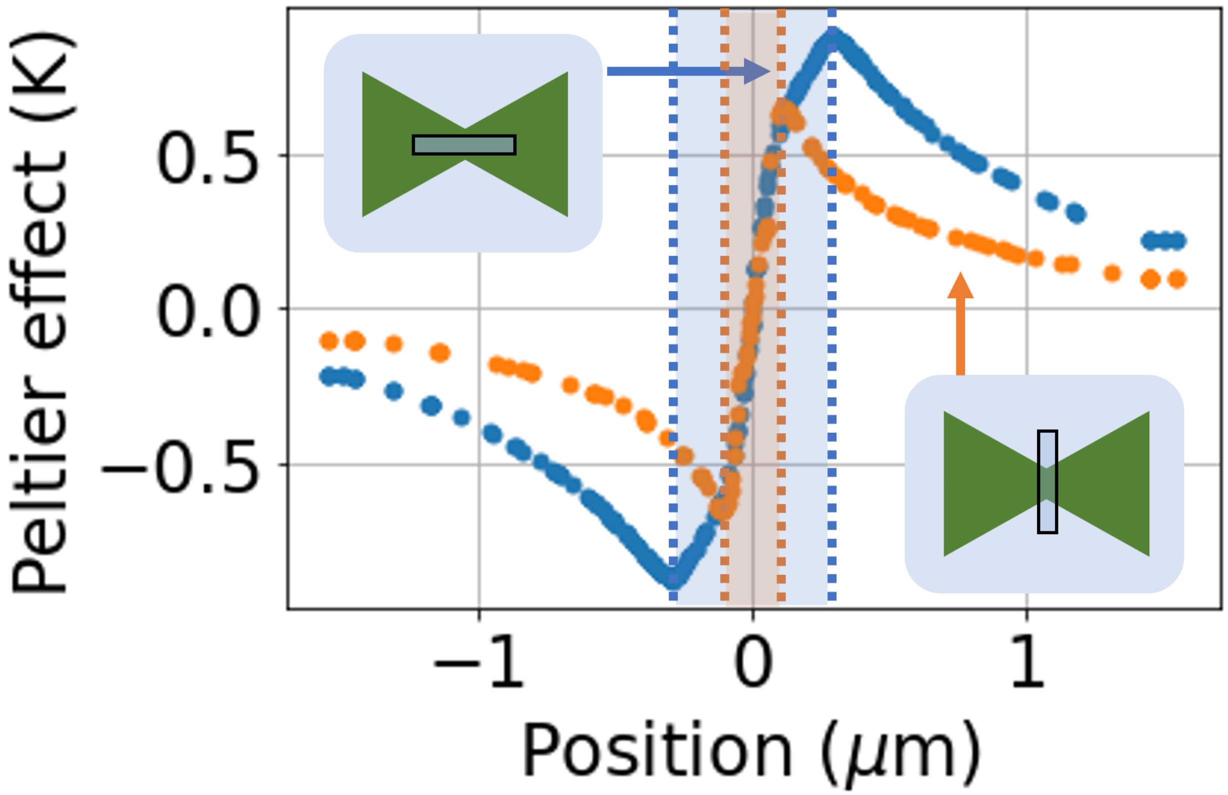

Figure S16: Simulation of the Peltier temperature for the aperture parallel (blue) and perpendicular (orange) to the channel direction. Schematic of each aperture direction is shown on the graph. The suspended part for both geometries is indicated by the shaded region.

6 Ballistic heat transport

To evaluate the heat transport mechanism in the suspended region of the device presented in Fig.2 (d) of the main text we model the temperature profile by assuming that the temperature rise is due to Joule heating and ignore the Peltier effect. The temperature profile is then given by[S6]

$$T(x) = \left(\frac{F}{2k}\right)(s - x)\,x + T_b, \tag{18}$$

where T_b is the temperature at the boundaries just after the jumps, s is the length of the device, k is the thermal conductivity of graphene and $F = PA^{-1}$ is the Joule heating generation term. By fitting Equation 18 to the experimental curve we get $k = 795Wm^{-1}K^{-1}$ for the suspended graphene, which is a reasonable value for suspended graphene.

The ratio of the temperature increase in the middle of the suspended region to the temperature jump at the contacts reveals information about the dominant heat transport mechanism,[S6]

$$\frac{\delta T}{\mid \Delta T \mid} = \frac{1}{8K_n/3}, \tag{19}$$

where $\delta T = T_\mathrm{b} - T_0$ with T_0 being the temperature at the boundaries before the jump, $\Delta T = T(x = s/2) - T_\mathrm{b}$ and $K_\mathrm{n} = \lambda/s$ is the Knudsen number given by the ratio of the phonon mean free path λ to the device length s. The Knudsen number is a measure of the heat transport regime; for diffusive transport λ is much smaller than s and $K_n << 1$ while for ballistic transport λ is larger than s and $K_n >> 1$. For quasi-ballistic transport $K_n \sim 1$. We find $K_n = 1.3$ indicating a quasi-ballistic phonon transport in the suspended graphene.

7 Additional Data

7.1 Resistance evolution

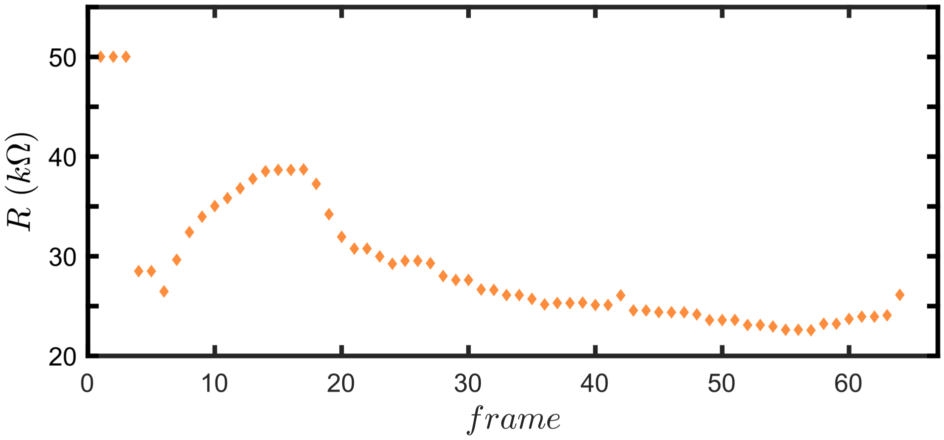

Figure S17: Resistance evolution of the graphene device with HAADF frames up to graphene breakdown, as presented in Figure 5 of the main text.

7.2 Additional devices

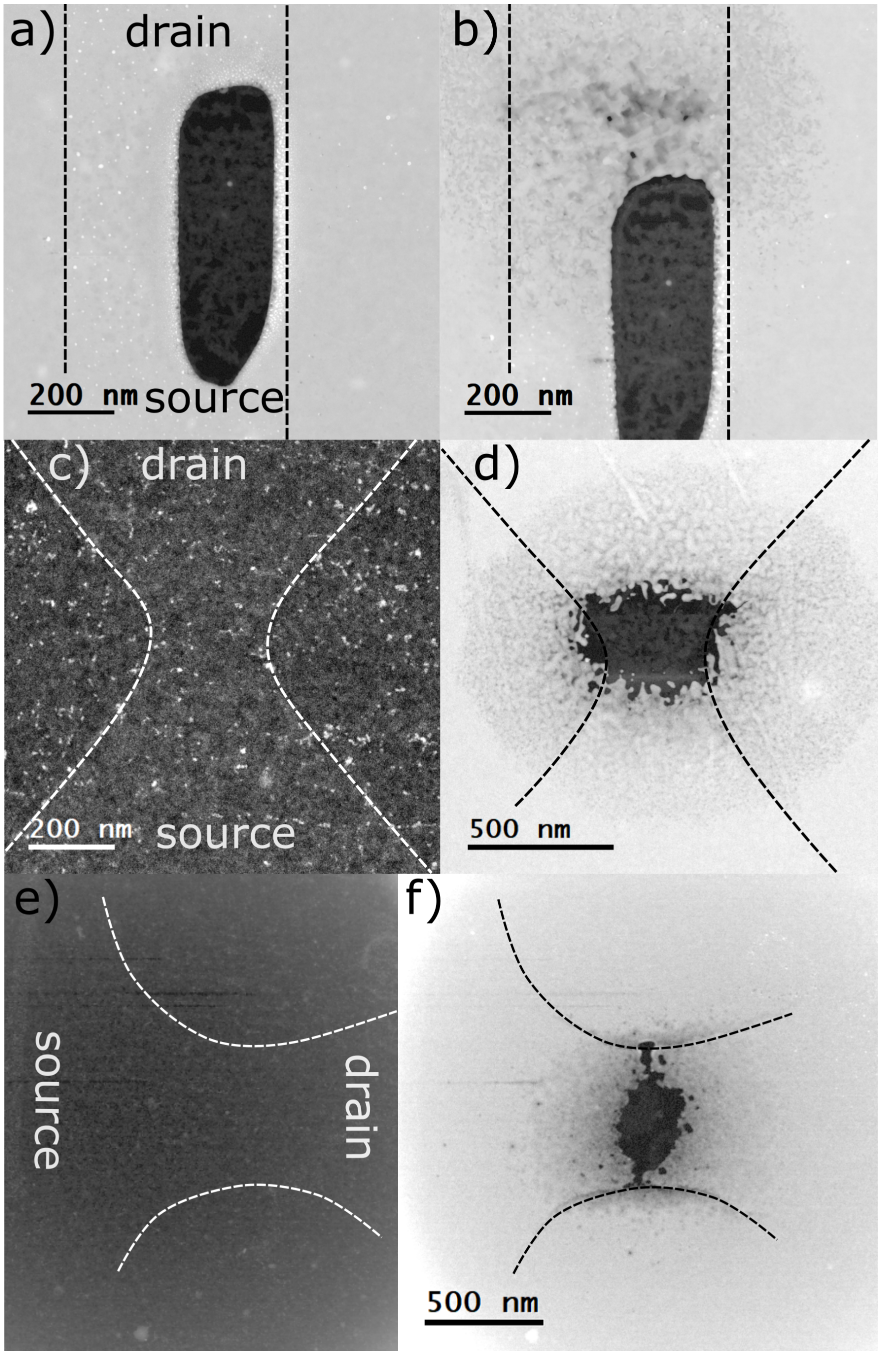

Figure S18: Examples of additional graphene devices (one suspended ribbon (a,b) and two bowtie supported (c-f)) before and after operation in high current density conditions. (a), (c), and (e) show HAADF images of each configuration prior to operation. (b), (d), and (f) show HAADF images of the devices after operation to failure.Graphene is shown with dotted lines. in all cases the damage of the substrate is shifted towards the drain side of the device.(f)(j-l) Adapted from Oxford University Press: Microscopy and Microanalysis ref.,[S7] copyright 2022.

References

(S1) Evangeli, C.; Spiece, J.; Sangtarash, S.; Molina-Mendoza, A. J.; Mucientes, M.; Mueller, T.; Lambert, C.; Sadeghi, H.; Kolosov, O. Nanoscale Thermal Transport in 2D Nanostructures from Cryogenic to Room Temperature. *Advanced Electronic Materials* **2019**, *5*, 1900331.

(S2) Spiece, J.; Evangeli, C.; Lulla, K.; Robson, A.; Robinson, B.; Kolosov, O. Improving accuracy of nanothermal measurements via spatially distributed scanning thermal microscope probes. *Journal of Applied Physics* **2018**, *124*, 015101.

(S3) Menges, F.; Riel, H.; Stemmer, A.; Gotsmann, B. Quantitative thermometry of nanoscale hot spots. *Nano letters* **2012**, *12*, 596–601.

(S4) Harzheim, A.; Spiece, J.; Evangeli, C.; McCann, E.; Falko, V.; Sheng, Y.; Warner, J. H.; Briggs, G. A. D.; Mol, J. A.; Gehring, P.; others Geometrically enhanced thermoelectric effects in graphene nanoconstrictions. *Nano letters* **2018**, *18*, 7719–7725.

(S5) Arganda-Carreras, I.; Kaynig, V.; Rueden, C.; Eliceiri, K. W.; Schindelin, J.; Cardona, A.; Sebastian Seung, H. Trainable Weka Segmentation: a machine learning tool for microscopy pixel classification. *Bioinformatics* **2017**, *33*, 2424–2426.

(S6) Kaiser, J.; Feng, T.; Maassen, J.; Wang, X.; Ruan, X.; Lundstrom, M. Thermal transport at the nanoscale: A Fourier's law vs. phonon Boltzmann equation study. *Journal of Applied Physics* **2017**, *121*, 044302.

(S7) Dyck, O.; Swett, J. L.; Evangeli, C.; Lupini, A. R.; Mol, J.; Jesse, S. Contrast Mechanisms in Secondary Electron e-Beam-Induced Current (SEEBIC) Imaging. *Microscopy and Microanalysis* **2022**, *28*, 1567–1583.
